# Temporal pairwise spike correlations fully capture single-neuron information

**DOI:** 10.1038/ncomms13805

**Published:** 2016-12-15

**Authors:** Amadeus Dettner, Sabrina Münzberg, Tatjana Tchumatchenko

**Affiliations:** 1 Theory of Neural Dynamics Group, Max Planck Institute for Brain Research, Max-von-Laue-Strasse 4, 60438 Frankfurt, Germany

## Abstract

To crack the neural code and read out the information neural spikes convey, it is essential to understand how the information is coded and how much of it is available for decoding. To this end, it is indispensable to derive from first principles a minimal set of spike features containing the complete information content of a neuron. Here we present such a complete set of coding features. We show that temporal pairwise spike correlations fully determine the information conveyed by a single spiking neuron with finite temporal memory and stationary spike statistics. We reveal that interspike interval temporal correlations, which are often neglected, can significantly change the total information. Our findings provide a conceptual link between numerous disparate observations and recommend shifting the focus of future studies from addressing firing rates to addressing pairwise spike correlation functions as the primary determinants of neural information.

## Abstract (web summary)

To understand the neural code it is important to determine what spiking features contain the relevant information. Here, the authors use mathematical approaches to show that two pair-wise correlation functions, the autocorrelation function within spike trains and cross-correlation function across stimulus presentations, fully determine the neural information content.

Throughout the central nervous system of a mammalian brain, spike times are the only events, which carry information about incoming stimuli, represent thoughts or drive motor behaviour. If we knew what features of a spike train contain all relevant information, then we could limit our attention to those features to extract the stimulus of interest or compare coding across regions. For example, if we knew that all stimulus information was contained in the second-order spike correlations, then we could focus our resources on obtaining exact estimates of these features to tell how much, if at all, can be learned about a stimulus from a spike train.

One would think that these basic questions are well understood, as experimental recording methods have been available for over a century and basic theories about communication channels, including Shannon's coding theory, appeared over 60 years ago. Surprisingly, many basic questions are still open. To understand the main difficulty, let us consider the set of spikes {*t*_*i*_} emitted by a neuron. Each of these spikes could occur at any time during the recording and therefore has infinite time resolution and potentially infinite information capacity. Infinite time resolution is typically overcome by using time binning where the spikes are assigned to one of *N* time intervals. In each time interval a neuron can either spike or remain silent, which results in 2^*N*^ possible spike patterns during the recording of *N* time bins. For a neuron recorded for 1 s at a 2 kHz sampling rate, there are billions of possible combinations. Some patterns could occur often across trials, others rarely; some could be coding relevant, whereas others could just be noise driven^1^,^2^. In general, the longer the recording, the more spikes are obtained and the bigger the combinatorial phase space becomes. This exponential expansion of the phase space is at the core of why neural coding is difficult to understand and information content equally difficult to estimate^3^. At the same time, growing experimental evidence offers a potential solution and indicates that neural activity does not explore all possible combinations but unfolds in many different brain regions along task-specific low-dimensional subregions^4^. To illustrate this typical coding situation in Fig. 1a, we sketch schematically a low-dimensional information coding sub-space on the background of the full phase space of all possible spiking combinations. As the dimensionality of the phase space of possible spike combinations increases, the information coding features can remain the same (see Fig. 1b).

What could these coding features be? Probably candidates put forward by previous studies include the average number of spikes per time^5^ or the occurrence frequency of spike doublets or triplets^6^. Currently, the most common and oldest approach to neural coding is the rate code hypothesis^5^, which postulates that the average number of spikes per time is the main carrier of information. The rate-coding hypothesis gave rise to a large body of literature describing how the average firing rates of neurons are modulated by different stimulus features such as animal location, stimulus orientation, motion, sound or light intensity^7^. Numerous studies, however, report that neurons can encode information without necessarily changing the average firing rate in response to a stimulus^5^,^8^,^9^. Yet so far, a durable and computationally tractable alternative to a rate code that curtails the combinatorial complexity of spiking activity has remained elusive.

Here we address the information content of a spike train and identify the minimal feature set that is sufficient and necessary for information coding. Motivated by the highly variable neural activity across time and repetitions^1^, and the observation of irregular but stable firing across time in many experiments^7^,^10^,^11^, we focused on the information coding in irregular, stationary spike trains with finite memory, which are supported by experimental *in vivo* evidence and are the cornerstone of current theoretical approaches^3^,^5^,^7^,^12^.

Surprisingly, our results show that information encoded in the spikes of a single neuron is fully described by only two pairwise spike features. The first feature is the pairwise autocorrelation function, which describes the coding-relevant temporal second-order interactions within a spike train. The second feature is the pairwise cross-correlation across noisy trials, which describes the coding-relevant aspects emerging from noise interactions. The cross-correlations are related to the temporal precision of the peaks in the peristimulus time histogram (PSTH) and may be known to some readers as the autocorrelation of the PSTH.

## Results

### Correlation theory of neural information

We are interested in the information contained in a spike train *r*(*t*) about a stimulus *s*(*t*), which is a part of this neuron's input current. We consider a situation where spike responses, as well as the currents that evoked them, are stationary in time and have finite temporal memory and finite, non-zero coefficient of variation. The spiking decision of the neuron is determined by its input current, which is a function of the stimulus *s*(*t*) and the noise *n*(*t*), with *s*(*t*) and *n*(*t*) being independent of each other. Stationary processes *r*(*t*),*s*(*t*),*n*(*t*) are characterized by a probability distribution such as *P*(*s*(*t*_1_),…*s*(*t*_*k*_)) that is invariant with respect to time translations^13^. In other words, their statistics depends neither on the start nor the end point of the recording and includes many prominent classes such as Markovian, non-Markovian, Gaussian or non-Gaussian time processes. The second property of finite memory guarantees that the interactions between any two recording times vanish if the two time points are sufficiently far apart. The assumption of finite memory is plausible for neurons, because any biological interaction has finite lifetime. The assumption of stationarity, which has already been the cornerstone of current theoretical approaches^3^,^7^,^10^,^11^,^12^, is motivated by the experimental evidence that the same stimulus presented at different time points produces similar outcomes^2^.

To express the information content we use the concept of mutual information, which is the difference between the signal and noise entropy^7^,^14^ and can be summarized via


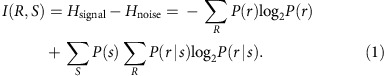


Here, *I*(*R*,*S*) is the mutual information between stimulus and response, *P*(*r*) is the probability distribution of all possible spike trains belonging to the set *R* resulting from varying stimulus presentations drawn from the set *S* and *P*(*r*|*s*) is the distribution of spike trains evoked by repeated presentations of stimulus *s*. This distribution, *P*(*r*|*s*), represents the residual, noise-induced variability for a given stimulus *s*^15^ and in our calculations stimulus and noise processes are independent of one another.

Shannon^14^ pointed out in his original article that mutual information, unlike entropies, is independent of the basis space used to define *P*(*r*) and *P*(*r*|*s*); therefore, any complete basis can be used to calculate the information. Let us note that the values of individual entropies are dependent on the coordinate system and may therefore vary across bases, methods and discretization procedures^16^. Taken individually, signal and noise entropies may therefore not be meaningful, yet their difference, the mutual information, is an invariant quantity that uniquely determines the information bandwidth of a neuron.

**Figure 2:**
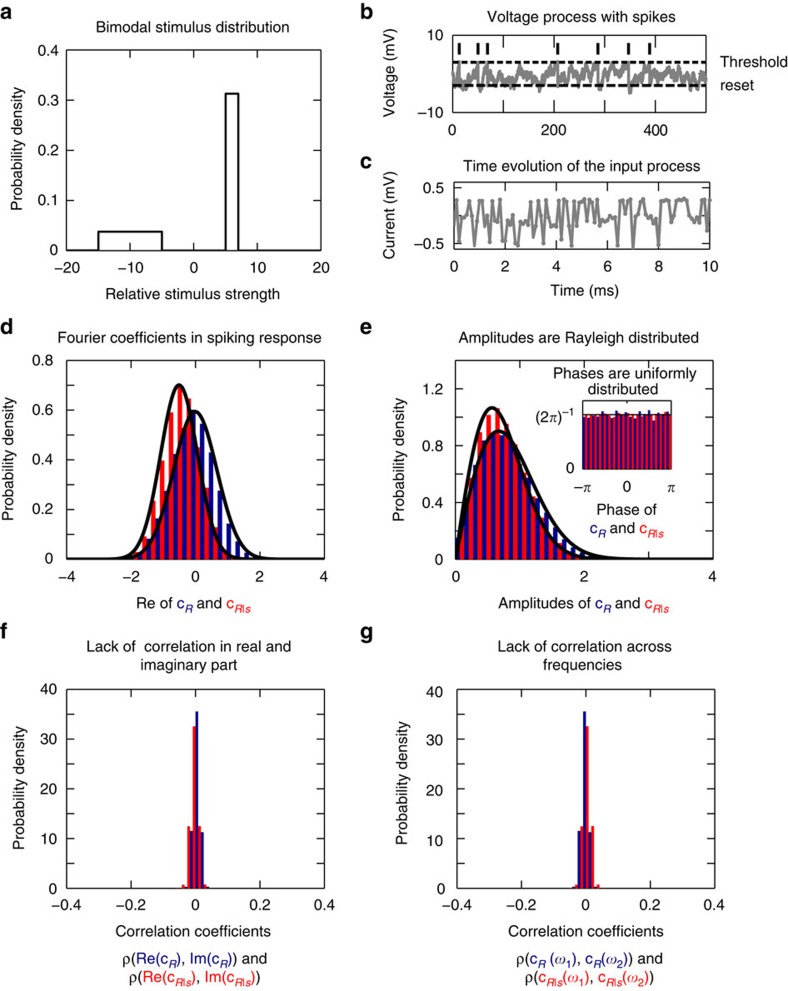
Distribution of the Fourier coefficients in a leaky integrate-and-fire neuron evoked by bimodal stimuli. (**a**) Bimodal stimulus distribution from which a stimulus value is drawn at each time step. (**b**,**c**) The resulting spike train of a leaky integrate-and-fire neuron and the underlying stimulus trajectory, respectively. Firing rate is 11.8 Hz and the corresponding mean interspike interval is 81 ms. (**d**) Probability distribution of Fourier coefficients *c*_*R*_(*ω*) (blue) and *c*_*R|s*_(*ω*) (red) from spiking responses in **c** for a frequency *f*=11 Hz (*ω*=2*πf*). It is noteworthy that the real part of Fourier coefficients *c*_*R*_ is zero mean (blue), whereas *c*_*R|s*_ have a non-zero mean (red). The non-zero mean does not affect the information content. (**e**) After the transformation of the mean-corrected Fourier coefficients to polar coordinates, we find that the amplitudes of *c*_*R*_ and *c*_*R|s*_ are Rayleigh distributed, whereas the phases are uniform (**e** inset). Black lines denote the respective fits. In **f** we confirm that the real and imaginary part for each frequency between 1 and 500 Hz are uncorrelated, as mathematical proofs indicate. (**g**) Similar lack of correlation holds across different frequencies (Δ*f*=1 Hz), whose residual non-zero values are due to finite recording size and decay with increasing recording time. Parameter values as in Table 1.

Following this idea, we choose the Fourier basis for probability calculations, because according to the work by Brillinger^13^ the distributions of Fourier coefficients across trials are mathematically very appealing. The Brillinger work indicates that even though the Fourier modes of a given spike train have highly structured phase and amplitude relationships across frequencies, the Fourier distributions across trials lack any complex structure in stationary, finite memory spike trains. Surprisingly, when they are pooled across trials independent, complex Gaussian distributions emerge. Let us stress that the reverse is not true, sampling independent Gaussian coefficients will not reproduce a spike train in the time domain, even if the mean and variance of the coefficients are matched. However, the fact that a spiking process is time-invariant and has finite memory, means that across trials these phase relationships are random. As shown in the Methods section, the Fourier coefficients for each frequency converge asymptotically towards independent complex normal distributions. For *P*(*r*), these normal distributions have zero mean, whereas those for *P*(*r*|*s*) have a non-zero mean (see Methods section and the Supplementary Note 1 for mathematical details). In Fig. 2, we confirm these properties in a leaky integrate-and-fire spiking model that is driven by a bimodal stimulus distribution. For each trial, we calculate the Fourier transform as described in the Supplementary Note 2. Figure 2a–c shows schematically the inputs and the resulting spikes. In Fig. 2d–g, we demonstrate the independence of Fourier coefficients and their Gaussian properties. In Fig. 2d,e, we plot the distribution of real values of the Fourier coefficient, its amplitude and phase at a single frequency *ω*=2*π*11 Hz across trials. We note that Fourier coefficients on trials with varying stimuli have zero mean, whereas trials with a repeating stimulus have a finite mean value, which we subtracted to obtain the Rayleigh distribution of amplitudes and uniform distribution of phases. Here, each of the Fourier coefficients for a frequency *ω=*2*πf* was obtained from 10^3^ trials each of length *T*=40 s. To explore the validity limits of these statistical properties, we consider a counter example and three limiting cases in the Supplementary Note 3 and in the Supplementary Figs 5–10.

As the mutual information for complex normal variables is determined only by the variances of 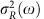 and 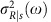, we can express these quantities and thereby the mutual information via the spike autocorrelation 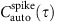 and spike cross correlation 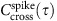 function:


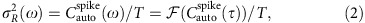



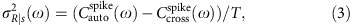


where 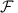 is the Fourier transformation and *T* the recording time. Here, *R* and *R|s* denote the sets of possible spike responses observed for varying or repeating stimulus presentations of *s*, respectively, where each *s*∈*S*. With these considerations, we now can express the full information rate using the spike correlation functions:


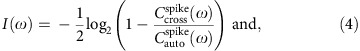



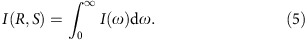


Here, *I*(*ω*) is the information rate per frequency in bit(s Hz)^−1^ and *I*(*R*,*S*) is the full mutual information rate transmitted by the spike trains in bit s^−1^ about a stimulus. We note that the spike cross-correlation function is identical to the autocorrelation of the PSTH, 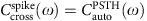 (see Methods).

In equation (5), we make two remarkable observations. Pairwise temporal spike correlations alone fully determine the complete stimulus information, whereas higher-order correlations between interspike intervals^17^ do not contribute additional information. Now, let us stress that we derived this result for a broad class of stationary and finite memory processes that apply to many but of course not all possible neural activity states. For example, some neurons may use phase coding^18^ or first latency coding^19^, which are beyond the validity limits of our correlation theory. In these cases, our theory can offer a quantitative benchmark for the efficiency comparison across coding schemes. To help relate our results to previous information theoretical approaches, we consider in the Methods section and the Supplementary Note 2 their relation to three frequently used approximate solutions, which include the lower bound estimation, the information carried by interspike intervals and the information carried by stimulus-induced rate variations.

### Novel insights into information coding

Here we use our correlation theory to explore the theoretical limits of information coding in a threshold-based spiking neuron^20^,^21^,^22^. We chose this particular model for two reasons. First, it captures the irregular spiking dynamics exhibited by L2/3 cortical pyramidal neurons^20^ that mediate long-range projections across the cortex. Second, it allows us to obtain exact information values with minimal numerical errors, as it offers exact, closed-form solutions for both auto- and cross-correlation functions^23^. This level of mathematical precision combined with biological relevance is currently not available in other integrate-and-fire-type models or experimental recordings and it allows us to precisely evaluate the influence of all possible variables on the neural information content. Model details can be found in the Methods section ‘Threshold-based neuron model'.

**Figure 3:**
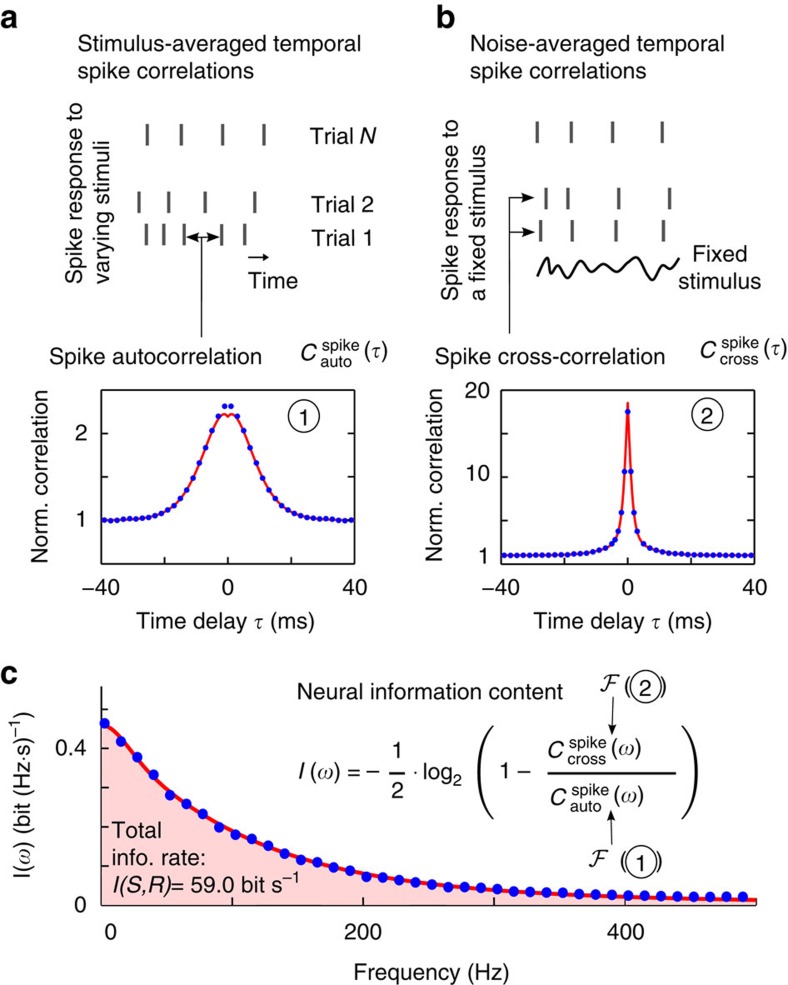
Correlation theory of neural information content. Analytical predictions (red) and numerical results (blue) for a threshold-based spiking model of pyramidal neurons^20^,^21^. The temporal spike correlations were obtained by calculating analytically (red line) and numerically (blue dots) the spike correlation functions (1) and (2). (**a**) Temporal correlations within a spike train are captured via the spike autocorrelation function. Noise variability across trials with repeating stimulus was captured via the spike cross-correlation function (**b**). By inserting the Fourier transformations (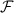) of the two functions (1) and (2) into equation (4), we obtain the exact information rate per frequency *I*(*ω*) and the total information rate of 59.0 bits *s*^−1^ shown in **c**. Parameter values as in Table 1. Spike correlation functions are normalized by the square of the firing rate to help compare auto- and cross-correlations.

By considering the spectral decomposition of information in this model, we found that for a neuron spiking at ∼8 Hz, only frequencies below 500 Hz contributed information. In the time domain, this translated to an informative spiking precision of up to 2 ms, which remarkably was two orders of magnitude smaller than the average interspike interval of ∼120 ms. Notably, this was in line with the ultrafast spike-detection kinetics reported for pyramidal neurons^24^, which has been observed but not yet related to a specific number of bits/spike conveyed in this frequency band. In addition, we found that the predicted information content agreed well with its numerical analogues (blue dots), which were obtained from spike trains (see Fig. 3c) with a finite duration of only 40 s, see Supplementary Note 2.

**Figure 4:**
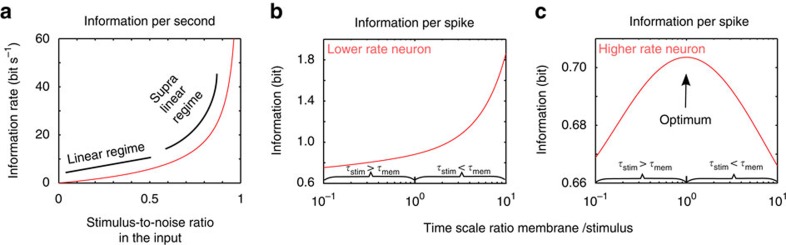
Nonlinear properties of neural information content. (**a**) The information rate per spike increases monotonically with input stimulus-to-noise ratio (SNR). In the low SNR limit, information gain is proportional to input SNR (linear regime). The steepness of the information gain grows for increasing SNR (supralinear regime). (**b**) In low-rate neurons (high spiking threshold), the information per spike grows with the membrane time constant. (**c**) Higher rate neurons (low threshold to variance ratio) exhibit a maximum in information transmission if the time scales of stimuli and membrane match each other, 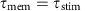. Parameter values as in Table 1.

We now took a further step and derived a number of novel predictions about the impact of input noise and neuronal excitability on the information coding capabilities of pyramidal neurons. First, we addressed the role of input noise by varying the stimulus amplitude relative to noise in the threshold-based model of pyramidal neurons. We hypothesized that a plausible strategy for a neuron to achieve a higher information throughput may be to decrease its noise and increase its stimulus-to-noise ratio (SNR). Indeed, Fig. 4a confirms that improving the SNR ratio of a neuron results in higher information content. Interestingly, we observed in the low SNR limit that the information content was proportional to the SNR. However, when we decreased the noise and reached higher SNR values, we observed that the information content accelerated and exhibited a supralinear growth as a function of SNR (see Fig. 4a). This finding is counterintuitive and implies that reducing noise in a noisy neuron would deliver returns in the decoding accuracy that are proportional to the reduction in noise. On the other hand, if the neuron is already reliable and has low noise, any additional noise reduction will give a disproportionally large information gain, as shown in Fig. 4a. This suggests a simple new operational principle: improving the most reliable neurons is better than combating noise in the noisiest neurons.

Addressing the role of excitability on information content we found that models of pyramidal neurons active at a low rate (high spiking threshold) can improve their information efficiency substantially by increasing their membrane time constant and thereby integrating more of the stimulus information into each of their spikes (see Fig. 4b). On the other hand, neurons spiking at a higher rate (low spiking threshold), would benefit more from matching the membrane integration time to the stimulus time scale (see Fig. 4c). This regime implies an operational advantage for neurons that match their membrane filter to the stimuli they receive, to code the stimulus information more precisely in the temporal correlations between spikes. In summary, our correlation theory allowed us to identify two new operational principles that depend on the noisiness of the neuron and its activity level. First, investments in noise reduction have the highest information pay-off in already reliable neurons. Second, investments in time scale matching between the intrinsic neuronal time constants have the highest information pay-off in high activity neurons. Our theoretical predictions put previous experimental reports of time scale matching^25^,^26^, as observed in some neurons, into a conceptual framework and explain why some neurons may be matching their membrane time scales and the resulting spiking time scales to the input they receive while others may not.

### Dissecting the constituents of neural information

The rate coding hypothesis^5^ postulates that the average number of spikes per unit time is the only variable carrying information. Numerous experimental recordings have shown that the modulation of firing rates can often be related to the presence or absence of certain stimulus features^7^. On the other hand, there is growing evidence that neurons encode stimulus features by changing the temporal structure of the spike train without changing the firing rate^5^,^8^,^9^. This temporal code could potentially have higher information coding capacity than the rate code. Using our results in equation (4) we can now disentangle the contribution of spike timing correlations from rate contribution. To this end, we consider two limiting cases. First, we compare the full information contained in a spike train with that of its Poissonian analogue, which has the same stimulus induced rate modulation (PSTH) as the original spike train, but which neglects all temporal interactions. Second, we compare the full information of a spike train with that contained in its interspike distribution *p*(ISI), where ISI is the interval between two successive spikes, see Supplementary Note 2 section ‘Information in interspike intervals'.

**Figure 5:**
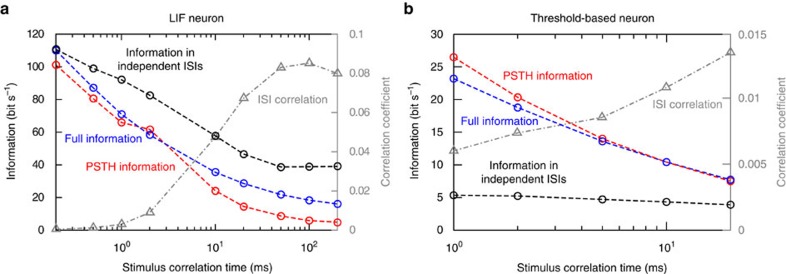
Temporal spike interactions impact neural information content. (**a**) The full information content predicted by the correlation theory (blue circles) along side the ISI information (black circles) and the Poisson rate approximation via PSTH (red circles) for a leaky integrate-and-fire neuron firing at 50 Hz. For reference, we also include in grey the correlation coefficient between successive interspike intervals. (**b**) The analogous quantities as in **a** for the threshold-based neuron firing at 5 Hz. Considering the PSTH-based Poisson rate approximation of information (red circles) results in an underestimation for slow stimuli in **a** and overestimation for fast stimuli in **b**. In **a**,**b**, we observe that temporal correlations can have two opposite effects, they can either increase the information by >50% (**a**) or decrease it (**b**) by a similar factor. To exclude firing rate effects as we vary time constants in **a**,**b**, we adjusted the spiking threshold to maintain a constant firing rate across all time constants. We further set 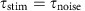, 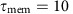
ms, other parameters in as in Table 1. Methods to ensure convergence of the ISI information can be found in the Supplementary Note 2 and Supplementary Fig. 4.

Addressing first the case of the Poissonian analogue, we find that the stimulus induced rate modulation is captured by the cross-correlation function, which appears in the numerator of equation (4) and which is equal to the autocorrelation function of the PSTH (see Methods section). Neglecting the temporal correlations replaces the original, temporally structured spike autocorrelation function with that of a Poisson spike train. In Fig. 5a,b, we show that considering only the rate covariations in the PSTH can result in both underestimation or overestimation.

To explore the role of temporal correlations we contrast the full information content with the information contained in the interspike interval distribution in the leaky integrate-and-fire model. Owing to a reset and the simple membrane filter, the range of spike correlations is at most as broad as the input's. As the input becomes more white the interspike correlations vanish quickly. We observe, as expected, a convergence between the ISI and the full information in the limit of small correlation times and vanishing interspike interval correlations in this model (see Fig. 5a). In the section ‘Information in the interspike intervals (ISI information)' of the Supplementary Note 2, we show that the main factor determining whether over- or underestimation occurs is the relative contribution of temporal effects and *p*(ISI) to 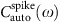. Here we demonstrate in Fig. 5 that both over- and underestimation can occur using two examples. Figure 5a demonstrates an overestimation example where the spiking follows the leaky integrate-and-fire model, firing rate 50 Hz and *τ*_men_=10 ms. Figure 5b demonstrates underestimation in the threshold-based model neuron, firing rate 5 Hz *τ*_mem_=10 ms. This model lacks a hard voltage reset and the spike times depend on the voltage derivative; therefore, its interspike correlations together with the deviations between the ISI and the full information may exist even for small input time constants.

### Advantages for experimental studies of neural information

Correlation functions have a long history in neuroscience, as they have been measured in live neurons and calculated in models for more than 50 years^27^,^28^. Our findings now demonstrate that they are vital ingredients for neural signal processing and can be used to calculate the full neural information content. With equation (4), we thus unite two previously disconnected fields of neuroscientific research—correlation studies and information theory.

Importantly, not only can previous correlation studies be revisited with regard to information but also our results will make future estimates of neural information more robust and easier to acquire. Here we argue that accessing information content via correlation functions has the potential to reduce the experimental data needs by at least two orders of magnitude compared with state-of-the art approaches.

**Figure 6:**
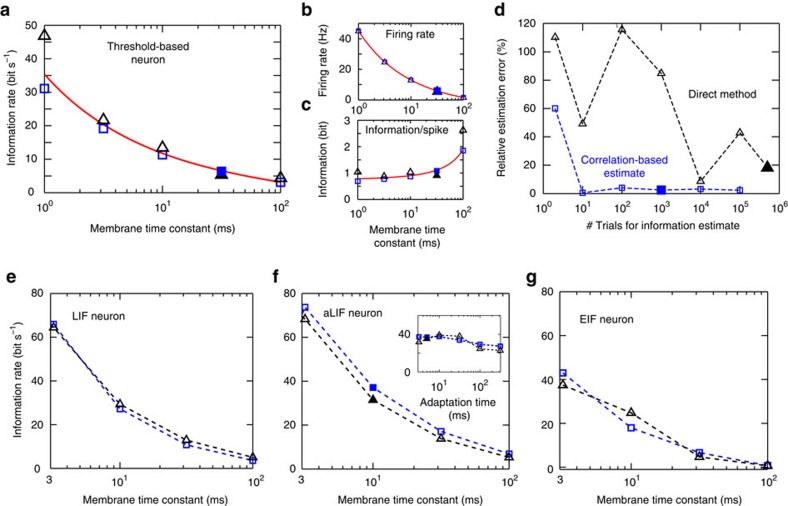
Correlation-based information estimates outperform the direct method. (**a**) Correlation-based information estimates (blue squares) and exact analytical values provided by the correlation theory (red line) agree with the direct method^16^ (black triangles) across two orders of membrane time constants in the threshold-based model. The corresponding firing rate and information per spike are in **b**,**c**, respectively. (**d**) Convergence to the exact information values for both correlation-based estimate and the direct method is shown for the selected data points (filled symbols) from **a**. We find that the correlation-based information estimate requires orders of magnitude smaller trial counts to achieve comparable accuracy and shows greater numerical stability. (**e**–**g**) We confirm the agreement between the correlation-based information estimate and the direct method in three additional spiking models: leaky integrate-and-fire neuron model (LIF, **e**), adaptive integrate-and-fire neuron model (aLIF, **f**) and the exponential integrate-and-fire model (EIF, **g**). (**f**) Inset: we vary the adaptation constant *τ*_ω_ across two orders in magnitude in the aLIF model, to demonstrate that our correlation theory applies to spike trains with complex internal structure that is not simply induced by the input. Parameter values as in Table 1 and spiking dynamics as described in the Methods section.

We show that the predictions of our correlation theory are consistent with previous information approaches^16^, as well as the information content reported *in vitro* and *in vivo*. Notably, our correlation theory meets these demands with a fraction of required data. In Fig. 6a–c, we show that the predictions of our correlation theory, its numerical implementation and the results obtained for the direct method^16^ align across two orders of magnitude of membrane time constants. Notably, the direct method needs at least two orders of magnitude longer recording lengths and at least five orders of magnitude larger trial numbers to meet the accuracy demands, see Fig. 6d. On the other hand, our correlation-based approach already converges with a small number of stimuli and exhibits substantially better numerical stability and low variability. This suggests that our results can significantly reduce the experimental recording lengths needed for an estimate of neural information content. Furthermore, we find that the predicted information range covering a few bits per spike agrees well with the 1.8 bits per spike observed in H1 neurons of the fly^16^, as well as the 0–3 bits s^−1^ observed in CA1 and CA3 regions of the hippocampus^29^. Details on the implementation and the numerical stability of the direct method can be found in the Supplementary Note 2. To verify that correlation theory and the direct method yield equivalent results across models, we evaluated both in three additional spiking models across two orders of magnitude in parameters. Figures 6e–g confirms that the correlation theory and the direct method yield equivalent results for the leaky integrate-and-fire model (Fig. 6e), for the adaptive integrate-and-fire model (Fig. 6f), as well as for the exponential integrate-and-fire model (Fig. 6g, see also Supplementary Figs 1–3) for an overview of the spiking statistics and a confirmation of complex Gaussianity in these models. Let us note, that in Fig. 6f we took particular care to demonstrate that not only stimulus induced but also intrinsically generated spike correlations can be captured by our correlation theory. To this end, we show in Fig. 6f (inset) correspondence between the correlation theory and the direct method in an adaptive integrate-and-fire neuron across two orders of adaptation time scales, which are comparable to experiment^30^,^31^,^32^. In this example, the stimulus and noise time scale is 20 ms, whereas the adaptation time scales range from 3 to 316 ms.

## Discussion

The list of spike timing features that have been implicated in neural coding includes the average number of spikes per time^5^ or the occurrence frequency of spike doublets or triplets^6^. Importantly, this list has experienced unprecedented growth in the last years as interactions between two, three or even *N* time points have increasingly been linked to neural information content^17^,^33^,^34^,^35^. In this study, we imposed fundamental limits on the growth of relevant coding features and showed mathematically that out of the infinitely large list of possible coding-relevant spike patterns, only two fully determined the neural information content in an important class of stationary neural codes with finite memory. These two features were the pairwise temporal spike correlation function within a spike train 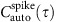 and the spike correlation function across repeated stimulus presentations 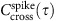, the latter of which may be known to some readers as the PSTH autocorrelation function, see Methods section.

The results we have presented here are independent of the spike generation details or neuronal type and apply to any experimentally recorded or simulated spike train that has finite memory and whose spiking statistics is time-invariant within the recorded time frame. In other words, our correlation theory showed that in neurons lacking an explicit time reference or ‘clock', the kind that is needed for example in phase coding^18^, the relative temporal correlations are the only functions that determine the information content. For the mathematically tractable threshold-based neuron model^20^,^21^ that is consistent with many features of cortical pyramidal neurons in L2/3, we used our correlation theory to construct the first exact value of information content. This allowed us to explore different operating regimes with an accuracy and speed that is beyond the reach of current experimental and numerical measurements.

A surprising aspect of neural coding became apparent when studying different SNR levels. We found that the SNR and information content are largely proportional to each other up to an SNR of ∼0.5, beyond which there is a supralinear increase in the information content (Fig. 4a). Investigating the contribution of excitability on neural information content, we found that for high spiking thresholds, neurons with a large membrane time constant have an operational advantage for transmitting information (Fig. 4b). On the other hand, more excitable neurons with a low spiking threshold tend to transmit information best in a narrow range of membrane time constants (Fig. 4c). The seminal work by Laughlin *et al*.^36^ and others suggested that neurons may be striving to find an optimal operating point to transmit the most information in the face of noise and energy^7^. Our work highlights how each of these constraints shapes the information transmission and provides a mathematically tractable platform to find the optimal operational point for any combination of input and neural excitability.

It has been proposed that most, if not all, neural information is carried in the average number of spikes per time rather than in the temporal spike patterns^7^,^19^. The accuracy of this rate code hypothesis has been repeatedly called into question^19^,^37^,^38^. Here we argued that the contribution of firing rate and temporal correlations are inextricable features of a correlation code. We showed in Fig. 5 that neglecting temporal correlations leads to significant errors in the information estimate. We also showed that calculating the information content under the assumption of independent interspike intervals can increase or decrease the information estimate by 50% or more. To identify the role of temporal rate variations, we related the PSTH to the trial cross-correlation function. We showed in Fig. 5 that this PSTH-based approximation is close to the complete information in some activity regimes, whereas in others this approximation can significantly deviate from the full information.

In addition, our correlation theory and especially equation (4) will significantly simplify the measurement of neural information in future studies. We have shown in Fig. 6 that the standard direct method^16^, which is now commonly used to estimate neural information, and the numerical implementation of our correlation theory agree well across two orders of parameter magnitudes in four different spiking models. However, what was remarkable is that to obtain comparable accuracy levels our correlation-based estimate required orders of magnitude lower trial numbers and recording lengths than the commonly used direct method and it showed improved numerical stability (Fig. 6d). Our correlation theory will thus reduce significantly the recording times for future experiments, improve numerical stability and, if closed-form expressions for correlation functions are available, provide an exact value of the information content.

To relate our results to previous information theoretical approaches, let us mention that we used insights from a carefully chosen basis transformation to eliminate the need to measure all possible spike patterns and instead suggest that it is sufficient to measure only those that contribute to two spike correlation functions. Although the direct method transformed spikes into binary words and provided a convergent numerical algorithm, it offered little guidance on how to reduce the word phase space to coding-relevant features^3^,^10^,^16^. Thus, at the core of both correlation theory and the direct method is the common idea induced by Grenander^39^ that a well-chosen basis transformation can be employed to make the probability calculations tractable and provide a more efficient probability estimation. This idea has already been used in a number of previous studies that attempted to numerically estimate probabilities, entropies and information in a variety of bases, which promised more attractive properties^3^,^10^,^16^,^40^. Yet, an open question remained from these studies with regard to two desirable features of the transformed basis space. First, it remained challenging to calculate interdependencies between bases projections and identify the most effective basis transformation. Second, providing exact solutions for all basis projections was often computationally demanding or required strong approximations, for example, Gaussian assumptions, and offered only a lower bound estimate^3^,^10^,^12^. We addressed these two properties in equations (1)–(5), 6, 7, 9, 10 and proved convergence and independence, while expressing the complete information via known pairwise correlation functions.

Now, let us comment on the potential generalizations of our correlation theory and opportunities for future studies. Using our correlation theory, we provided closed-form expressions for the quantity of mutual information, which can now be generalized to other information theoretical quantities using the work by Brunel and Nadal^41^. This group has shown that information theoretical quantities such as mutual information, Fisher information and readouts are intrinsically intertwined, such that mutual information can be transformed into Fisher information, which in turn directly determines the Cramer–Rao bound on readout accuracy^41^,^42^. Thus, the pairwise spike correlation functions can be used to derive an explicit limit on readout accuracy. In addition, our results can be used to shed light on the information content in recurrent networks. In this study, we have focused on the information content in individual neurons—the constituents of a neural network. In a recurrent network, where the dynamics of each neuron satisfies the stationarity and finite memory conditions, our theory can be applied to each constituent neuron. Considering the sum across all neurons we were able to obtain a first-order approximation of how features such as firing rates, time scales and noise levels have an impact on network level coding. Future studies could also address specific connectivity scenarios where our correlation theory generalizes to *N* dimensions and where coupling between Fourier coefficients across neurons could reveal novel network coding strategies. Probable candidates for networks amenable to our theory are irregular, balanced neural networks, where each neuron has a finite correlation time^43^. Extending our correlation theory to interneuronal interactions in recurrent networks could provide a mechanistic understanding of the information carrying features in networks and connect to existing Ising-type models describing cortical and retinal activity^44^. Identifying the contributions of individual neurons and that of synaptic interactions could help reveal the quantitative determinants of network information coding.

## Methods

### Deriving the correlation theory of neural information

We are interested in the information contained in the spike train *r*(*t*) about a stimulus *s*(*t*). The spike train is given by a sum of delta functions 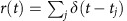 where *t*_*j*_ are spike times. The stimulus can be any time-continuous or discrete stochastic process. To mathematically formalize the information contained in *r*(*t*) about *s*(*t*), we use the concept of mutual information, which is given by the difference between signal and noise entropies 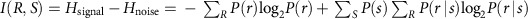. We denote by *R* and *R*|*s* the sets of possible spike responses observed for varying or repeated stimulus presentations *s*, respectively, where each *s* is taken from the set 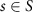. The results derived in the following are valid for recorded neurons and spiking neuron models that fulfill the following assumption: spike trains *r*(*t*), the underlying stimulus *s*(*t*) and the noise *n*(*t*) are each stationary random processes with finite memory and finite mean and variance (see Supplementary Note 1 for more formal definitions). Let us note that the assumption of finite memory is plausible for any biological system, because ion channels, proteins or any other biological molecules have finite lifetimes. The stationarity condition can be fulfilled by any neuron whose spiking mechanism remains constant during the recording period and which therefore responds with the same statistics to current trajectories regardless of whether they are presented at the beginning or the end of a recording. This can be fulfilled by a diverse set of spiking mechanisms that may include spike-triggered or subthreshold adaptation, or have a threshold-based spike condition. To highlight that our theory is valid across spiking models, we demonstrate its validity using the following spiking mechanisms: leaky integrate-and-fire, adaptive integrate-and-fire and the exponential integrate-and-fire spiking mechanisms (see Methods sections below). To explicitly calculate the mutual information, we use the statement in the original paper by Shannon (p.42, part IV in ref. 14) that the mutual information is a basis-independent quantity. This statement allows us to exchange the time domain for a more convenient basis. Specifically, we search for a basis space that simplifies the probability summation and endows the information evaluation with more attractive statistical properties. To this end, we choose the Fourier basis, because it has been shown by Brillinger (see p.94, Theorem 4.4.1 in ref. 13 and refs 45, 46 for extensions to point processes) to offer statistically independent basis projections (principal components) that are mathematically highly tractable.

We now proceed to define the new basis projections, which are the Fourier coefficients 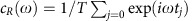, 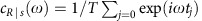 and study their distributions over many trials of the same duration. The results of Brillinger (see p.94, Theorem 4.4.1 in ref. 13) imply that 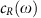 and 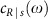 asymptotically approach a complex normal distribution. *c*_*R*_(*ω*) is a complex normal distribution with zero mean and finite variance, whereas our calculations indicate a finite mean for 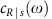 (see Supplementary Note 1). Let us briefly note that the complex normal distribution is derived from the Central Limit Theorem applied to the quantity 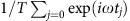. Knowing that spike times *t*_*j*_ are correlated only in a finite time window implies that spike trains exceeding a multiple of this window will contribute largely uncorrelated spikes to this sum and thereby lead to a normal distribution. As the variances of *c*_*R*_(*ω*) and 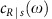 are the only quantities determining the information content for each Fourier mode, we proceeded to derive them from the spike train statistics and obtain 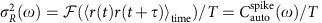, 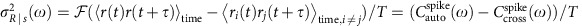. We can now proceed and simply integrate the contributions of the frequencies because each is statistically independent according to the proofs by Brillinger^13^, as we detail in the Supplementary Note 1.

To obtain the contribution of each individual Fourier mode to the complete information content, we need to evaluate each mode's amplitude and phase distribution. Specifically, we have to ensure that amplitude and phase (or real and imaginary values) are contributing non-redundant information. Using the auxiliary calculations detailed in the Supplementary Note 1, we show that the amplitude of 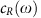 follows a Rayleigh distribution with variance 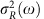, while its phase is uniformly distributed^47^. Analogously, the amplitude of the mean-corrected coefficient 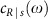 follows a Rayleigh distribution with variance 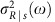. As the entropies of complex normal distribution with zero and non-zero means have the same entropy, we zero-centre the normal distribution of 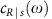 by subtracting its mean. We are now faced for each Fourier coefficient with a Rayleigh distribution describing the amplitude and a uniform distribution describing the phase, see also Fig. 2 for a numerical demonstration. For both Fourier coefficients 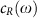 and 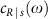, the corresponding Rayleigh and the uniform distributions are statistically independent of each other. Combining these insights we find that the neuronal information is carried only by the Rayleigh-distributed amplitudes, and that the uniform phase distribution carries no additional information. Thus, we can proceed to evaluate the complete information by considering that 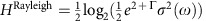, where Γ is the Euler–Mascheroni constant. We obtain:


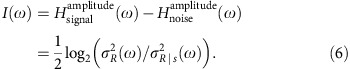


Using the fact that 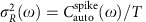 and 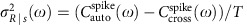, we obtain the result in equation (5).

### Relating correlation theory to the lower bound estimation

To link our results to previous approaches that have been derived for Gaussian stimuli and Gaussian response statistics by Rieke *et al*.^11^, Bialek *et al*.^10^ and others, we show that from our general result we can recover the coherence-based lower bound on information content. Starting from equations (4) and (5), we Taylor expand the spike cross-correlation function in the variable SNR ratio and consider only its first order kernel 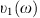
49 (for details see Supplementary Note 1). We obtain


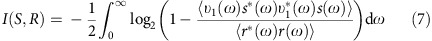



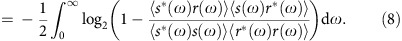


This result is based on the linear approximation of the full spike cross-correlation function and is naturally a lower bound on the information content. We recognize, however, that this linearization procedure is not limited to the Gaussian stimuli even though it was originally derived via the Wiener kernel expansion for Gaussian inputs^10^,^11^,^12^,^48^.

### Information in stimulus-induced rate variations

To estimate the information contained in the stimulus-induced rate variations, we consider the PSTH measured in trials with repeated stimulus presentation. To this end, we derive a relation between PSTH and the pairwise cross-correlation function measured across trials. Following the calculations detailed in the Supplementary Note 1, we show that in the limit of infinitely long recording lengths *T* the autocorrelation of the PSTH 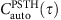 corresponds to the pairwise cross-correlation function 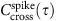. We obtain


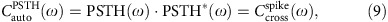


where 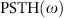 is the Fourier transform of the PSTH and 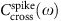 is the Fourier transform of 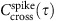. Considering trials with a varying stimulus, we obtain a flat autocorrelation function, because any temporal structure present in individual spike trains is averaged out across trials. Thus, neglecting intrinsic temporal structure within each spike train results in a Poisson-like flat autocorrelation function. In this situation the neural information content is determined by


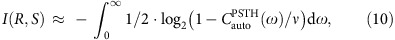


where *v* is the firing rate across trials. In Fig. 5, we compare this approximation with the complete information content.

### Input current statistics

Here we define the statistics of input currents, which we use in this study. The input current *X*(*t*) is a weighted sum of a stimulus *s*(*t*) and a noise process *n*(*t*), and satisfies the equation 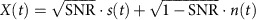, where SNR is the stimulus-to-noise ratio, SNR∈[0,1), and *s*(*t*) and *n*(*t*) are statistically independent processes. The statistics of both *s*(*t*) and *n*(*t*) follows either Ornstein–Uhlenbeck Gaussian process^50^ (Figs 3, 4, 5, 6 and Supplementary Figs 1–3) or a bimodal distribution in (Fig. 2). The differential equation defining the Ornstein–Uhlenbeck process *x*(*t*), where *x*(*t*) is either *s*(*t*) or *n*(*t*) is 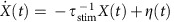, whereby *η*(*t*) is a zero mean white noise process with variance *σ*_*η*_ and *τ*_stim_ is its time constant. For mathematical tractability, we choose zero mean Ornstein–Uhlenbeck stimuli *s*(*t*) and *n*(*t*) to have the same variance *σ*_*η*_ and correlation time constant *τ*_stim_ between 1 and 50 ms, which corresponds to AMPA and NMDA time scales within a recurrent network (see Table 1). For bimodal stimuli, *s*(*t*) and *n*(*t*) each is independently drawn at each time step from the bimodal distribution in Fig. 2a. We note that both Ornstein–Uhlenbeck and bimodal currents are stationary and have finite memory and by combining this with the neural dynamics described below, we obtain spike trains that also retain these features and lead to Gaussian Fourier coefficients that are independent across frequencies, see Fig. 2.

### Spiking neuron models

In the following Methods section we present four spiking neuron models, which we used in Figs 2, 3, 4, 5, 6 and for which we detailed the Fourier statistics in the Supplementary Figs 1–3. All four spiking models were chosen based on their relevance to the dynamics of live neurons. We chose the threshold-based neuron model^20^,^21^,^22^ (Figs 4, 5, 6) based on its similarity to pyramidal L2/3 neurons in the visual cortex^20^ and its mathematical tractability^23^. The leaky integrate-and-fire, adaptive leaky integrate-and-fire, as well as exponential integrate-and-fire neuron models in Figs 2, 5a and 6 were chosen due to their similarity to pyramidal neurons in L4 and L2/3 (refs 7, 51, 52, 53) and cortical neurons exhibiting subthreshold oscillations^54^.

### Threshold-based neuron model

In this model, the voltage dynamics are governed by^20^,^21^:


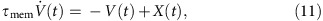


where *V*(*t*) is the membrane voltage and *τ*_mem_ is the membrane time constant. *X*(*t*) is the zero mean input current, whose temporal evolution follows an Ornstein–Uhlenbeck process^50^ described by the differential equation 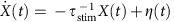, whereby *η*(*t*) is a zero mean white noise process with an s.d. *σ*_*η*_ and time constant *τ*_stim_. The input current *X*(*t*) incorporates input resistance and carries the unit mV. The input current consists of a mixture 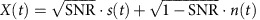, where SNR is the stimulus-to-noise ratio, and both *s*(*t*) and *n*(*t*) share the same *τ*_stim_ and *σ*_*η*_. The neuron emits a spike if a voltage threshold *V*_th_ is crossed from below. This model can be exactly mapped to the integrate-and-fire model for small firing rates and finite time constants (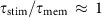)^22^. The model's main computational advantage relative to the leaky integrate-and-fire model is its mathematical tractability^23^. We note that by considering noise and stimuli, which both have zero mean, as we do throughout this study, the voltage will also have a zero mean and this neuron model will only be able to reach the threshold and emit a spike if either the temporally varying noise or the stimulus is present. This situation corresponds to the subthreshold regime introduced by Gerstner and Kistler^19^. In Fig. 3 we set *σ*_*η*_=0.45 mV and *V*_th_=0.6; in Fig. 5b we set *σ*_*η*_=1 mV, and for *τ*_stim_=[1,2,5,10,20] ms we set the thresholds to *V*_th_=[0.46,0.81,1.58,2.41,3.29] mV. In Fig. 6 we set *σ*_*η*_=*V*_th_=1 mV, to achieve the best comparison with other integrate-and-fire models. All other parameter values are given in Table 1.

### Leaky integrate-and-fire neuron model

In this model, the voltage dynamics are governed by^7^:


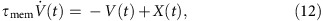


where *V*(*t*) is the membrane voltage, *τ*_mem_ is the membrane time constant and *X*(*t*) denotes the input current. The input current *X*(*t*) has either a bimodal distribution as in Fig. 2 or is a zero mean Ornstein–Uhlenbeck process^50^ with a time constant *τ*_stim_. In the latter case, the temporal evolution of *X*(*t*) is described by the differential equation 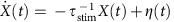, whereby *η*(*t*) is a zero mean white noise process with an s.d. *σ*_*η*_ and time constant *τ*_stim_. The input current *X*(*t*) incorporates input resistance and carries the unit mV. The input current consists of a mixture 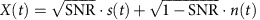, where SNR is the stimulus-to-noise ratio, and *s*(*t*) and *n*(*t*) have the same statistical properties. For example, when we consider Ornstein–Uhlenbeck inputs, both *s*(*t*) and *n*(*t*) have the same *τ*_stim_ and the same *σ*_*η*_. The same applies to bimodal inputs, in which case both *n*(*t*) and *s*(*t*) are drawn from the same bimodal distribution. In this model, the neuron emits a spike whenever the voltage *V*(*t*) reaches a threshold value *V*_th_, after which the voltage is reset to *V*_reset_. For Ornstein–Uhlenbeck inputs we choose *σ*_*η*_=1 mV and *V*_th_=−*V*_reset_=1 mV. In Fig. 5a for *τ*_stim_=[0.2,0.5,1,2,10,20,50,100,200] ms, the thresholds are *V*_th_=−*V*_reset_=[0.03,0.06,0.12,0.21,0.65,1.01,1.69,2.45] mV. For bimodal inputs we set the threshold and reset values to *V*_th_=−*V*_reset_=3 mV, to achieve a biologically realistic firing rate. We note that by considering Ornstein or bimodal noise and stimuli, which both have zero mean, as we do throughout this study, the voltage will also have a zero mean and this neuron model will only be able to reach the threshold and emit a spike if either the temporally varying noise or the stimulus are present in the input current *X*(*t*). This situation corresponds to the subthreshold regime introduced by Gerstner and Kistler^19^. For an illustration of the input and spike statistics, see Supplementary Fig. 1. All other parameter values are as in Table 1.

### Adaptive leaky integrate-and-fire neuron model

This spiking model is characterized by a subthreshold frequency preference and integrate-and-fire-type dynamics. The voltage dynamics in this model are governed by^53^,^54^:


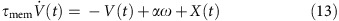



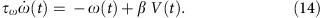


Here, *V*(*t*) is the membrane voltage, *τ*_mem_ is the membrane time constant and *α*, *β* and *τ*_*ω*_ are adaptation variables. *X*(*t*) is the input current, whose temporal evolution is described by the differential equation 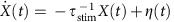, whereby *η*(*t*) is a zero mean white noise process with an s.d. *σ*_*η*_ and time constant *τ*_stim_. The input current *X*(*t*) incorporates input resistance and carries the unit mV. The input current consists of a mixture 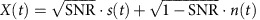, where SNR is the stimulus-to-noise ratio, and both *s*(*t*) and *n*(*t*) share the same *τ*_stim_ and *σ*_*η*_. In this model, the neuron emits a spike whenever the voltage *V*(*t*) reaches a threshold value *V*_th_, after which the voltage is reset to *V*_reset_. We note that by considering noise and stimuli, which both have zero mean, as we do throughout this study, the voltage will also have a zero mean and this neuron model will only be able to reach the threshold and emit a spike if either the temporally varying noise or the stimulus is present. This situation corresponds to the subthreshold regime introduced by Gerstner and Kistler^19^. Throughout our study we set the adaptive coupling constants as *α*=−2 and *β*=4. In Fig. 6f, we set *σ*_*η*_=1 mV, *V*_th_=0.7 mV, the reset voltage *V*_reset_=−1 mV and *τ*_*ω*_=5 ms. For illustration of the spiking statistics in Fig. 6f, see Supplementary Fig. 2. In the inset to Fig. 6f, the membrane constant is set at *τ*_mem_=10 ms, whereas *τ*_*ω*_ is varied. In this figure, the firing rate is kept at 50 Hz by adapting the threshold *V*_th_ and setting the reset value to *V*_reset_=−1 mV. Specifically, for *τ*_*ω*_=[3.2,5,10,31,100,316] ms the thresholds are *V*_th_=[0.63,0.7,0.82,1.09,1.4,1.61] mV; all other parameter values are as in Table 1.

### Exponential integrate-and-fire neuron model

In this model, the voltage dynamics are governed by^52^


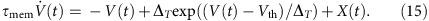


Here, *V*(*t*) is the membrane voltage and *τ*_mem_ is the membrane time constant. *X*(*t*) is the input current, whose temporal evolution is described by the differential equation 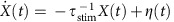, whereby *η*(*t*) is a zero mean white noise process with an s.d. *σ*_*η*_ and time constant *τ*_stim_. The input current *X*(*t*) incorporates input resistance and carries the unit mV. The input current consists of a mixture 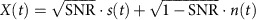, where SNR is the stimulus-to-noise ratio, and both *s*(*t*) and *n*(*t*) share the same *τ*_stim_ and *σ*_*η*_=1 mV. In this model, the neuron emits a spike whenever the voltage *V*(*t*) reaches a threshold value *V*_th_=1.25 mV, after which the voltage is reset to *V*_reset_=−1.25 mV. Δ_*T*_=1/2 mV is the slope factor determining the speed of spike initiation. All other parameter values are as in Table 1.We note that by considering noise and stimuli, which both have zero mean, as we do throughout this study, the voltage will also have a zero mean and this neuron model will only be able to reach the threshold and emit a spike if either the temporally varying noise or the stimulus is present. This situation corresponds to the subthreshold regime introduced by Gerstner and Kistler^19^. For an illustration of the input and spike statistics see Supplementary Fig. 3.

### Code availability

The computer code used in this study is available from www.tchumatchenko.de/Code_SNArticle.zip

### Data availability

Data sharing not applicable to this article, as no data sets were analysed during the current study. All results are either theoretical in nature or were obtained using the computer code above.

## Additional information

**How to cite this article:** Dettner, A. *et al*. Temporal pairwise spike correlations fully capture single-neuron information. *Nat. Commun.* **7,** 13805 doi: 10.1038/ncomms13805 (2016).

**Publisher's note:** Springer Nature remains neutral with regard to jurisdictional claims in published maps and institutional affiliations.

## Supplementary Material

Supplementary Information Supplementary Figures, Supplementary Table, Supplementary Notes and Supplementary References

Supplementary Software This supplementary file contains computer code underlying the results of the manuscript and it includes an implementation of the correlation-based information estimate, ISI-based information and the direct method. Within we provide also an additional file examplecode-fcreconstruction.zip to help understand how Gaussian Fourier statistics can emerge in stationary spike trains when Fourier coefficients are pooled across multiple trials.

## Figures and Tables

**Figure 1:**
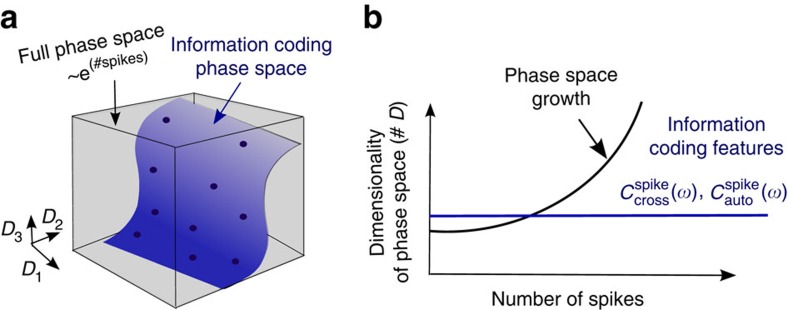
Dimensionality of neural information coding. (**a**) Phase space spanned by possible spike time combinations (grey box) relative to the information coding subspace (blue area) for a given number of spikes. If the relevant features are known, only the lower dimensional subspace has to be sampled (black dots), to estimate the neural information content. (**b**) Phase space grows exponentially with increasing number of spikes, whereas the relevant coding features remain constant. In this study we argue that the information-relevant features are 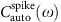 and 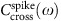, which we derive in equations (2) and (3).

**Table 1:** Parameter sets across neuron models.

Figure	*>τ*_stim_, *τ*_noise_	SNR	Membrane properties	Firing	Spiking neuron	Input
number	(ms)		(ms)	rate (Hz)	model	type
2	−	0.5	*τ*_mem_=10	11.8	LIF	Bimodal
3	3	0.8	*τ*_mem_=5	8.3	TB	OU
4a	10	0–1	*τ*_mem_=20	8.3	TB	OU
4b, 6a–d	10	0.6	*τ*_mem_=1–100	1.7–45	TB	OU
4c	10	0.6	*τ*_mem_=1–100	5–50	TB	OU
5a	0.2–200	0.6	*τ*_mem_=10	50	LIF	OU
5b	1–20	0.6	*τ*_mem_=10	5	TB	OU
6e	10	0.6	*τ*_mem_=3.2–100	1.1–107	LIF	OU
6f	20	0.6	*τ*_mem_=3.2–100	1.0–200	aLIF	OU
6f, inset	20	0.6	*τ*_mem_=10	50	aLIF	OU
			*τ*_ω_=3.5-316			
6g	10	0.5	*τ*_mem_=3.2–100	0.9–91	EIF	OU

aLIF, adaptive leaky integrate-and-fire; Bimodal, bimodal inputs; EIF, exponential integrate-and-fire; LIF, leaky integrate-and-fire; OU, Ornstein-Uhlenbeck processes; SNR, stimulus-to-noise ratio; TB, threshold based.

In this study, we investigated a TB, an LIF, an aLIF and an EIF model neuron. The input currents were OU processes or Bimodal.
