# Supplementary material for: Temporal pairwise spike correlations fully capture single-neuron information
Source: Nat Commun. 2016 Dec 15;7:13805. doi: 10.1038/ncomms13805 (PMC5171810; doi:10.1038/ncomms13805)
Supplement: Supplementary Information — Supplementary Figures, Supplementary Table, Supplementary Notes and Supplementary References [file ncomms13805-s1.pdf]

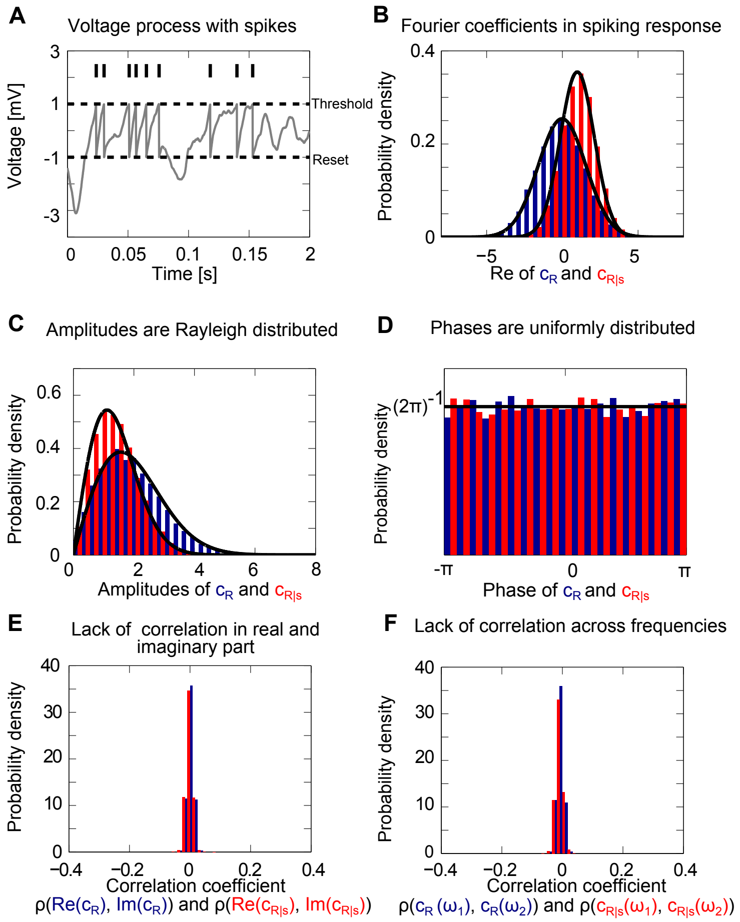

Supplementary Figure 1: Fourier statistics of leaky integrate-and-fire spiking responses (LIF) evoked by Ornstein-Uhlenbeck stimuli from Fig. 6 E in the main article. (**A**) Voltage trajectory and spikes in a LIF spiking neuron. In this model a spike (black vertical line) is emitted whenever the voltage crosses a threshold value, after a spike voltage is reset to its reset value. Here, the firing rate is 29 Hz and the mean interspike interval is 33 ms. (**B**) Probability distribution of Fourier coefficients $c_R(\omega)$ (blue) and $c_{R|s}(\omega)$ (red) in spiking responses from (A). (C) We can confirm that the amplitudes are Rayleigh-distributed and that the phases are uniformly distributed (**D**). Black lines in (C) and (D) denote the respective fits. In (**E**) we numerically confirm that the real and imaginary part for each frequency between 1 and 500 Hz are indeed uncorrelated, as mathematical proofs indicate. (**F**) Similar lack of correlation holds across different frequencies ($\Delta f = 1$ Hz). We note that the deviation of the mean correlation coefficient in (E) and (F) is a finite size effect that can be further reduced by increasing the recording duration and trial number. In this figure we present Fourier statistics at $\omega = 22\pi$ Hz, $\tau_{mem} = 10$ ms, analogous statistics can be observed at other frequencies and membrane time constants.

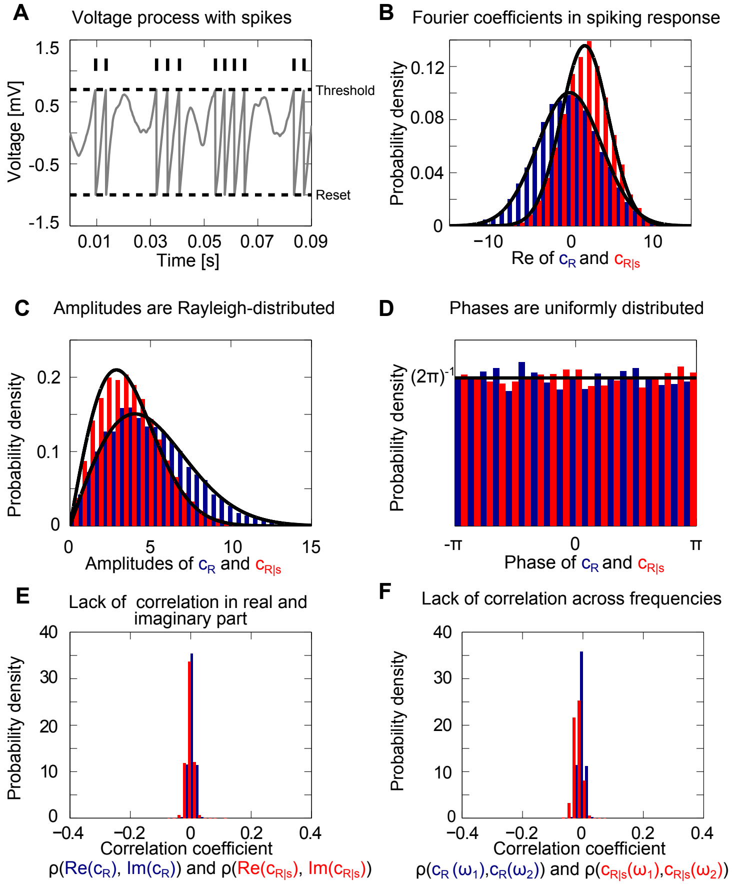

Supplementary Figure 2: Fourier statistics of adaptive leaky integrate-and-fire spiking responses (aLIF) evoked by Ornstein-Uhlenbeck stimuli from Fig. 6 F in the main article. (**A**) Voltage trajectory and spikes in a aLIF spiking neuron. In this model a spike (black vertical line) is emitted whenever the voltage crosses a threshold value, after a spike voltage is reset to its reset value. Here, the resulting firing rate is 50 Hz and the mean interspike interval is 19 ms. (**B**) Probability distribution of Fourier coefficients $c_R(\omega)$ (blue) and $c_{R|s}(\omega)$ (red) in spiking responses from (A). (C) We can confirm that the amplitudes are Rayleigh-distributed and that the phases are uniformly distributed (**D**). Black lines in (C) and (D) denote the respective fits. In (**E**) we numerically confirm that the real and imaginary part for each frequency between 1 and 500 Hz are indeed uncorrelated, as mathematical proofs indicate. (**F**) Similar lack of correlation holds across different frequencies ($\Delta f = 1$ Hz). We note that the deviation of the mean correlation coefficient in (E) and (F) is a finite size effect that can be further reduced by increasing the recording duration and trial number. In this figure we present Fourier statistics at $\omega = 8\pi$ Hz, $\tau_{mem} = 10$ ms, analogous statistics can be observed at other frequencies and membrane time constants.

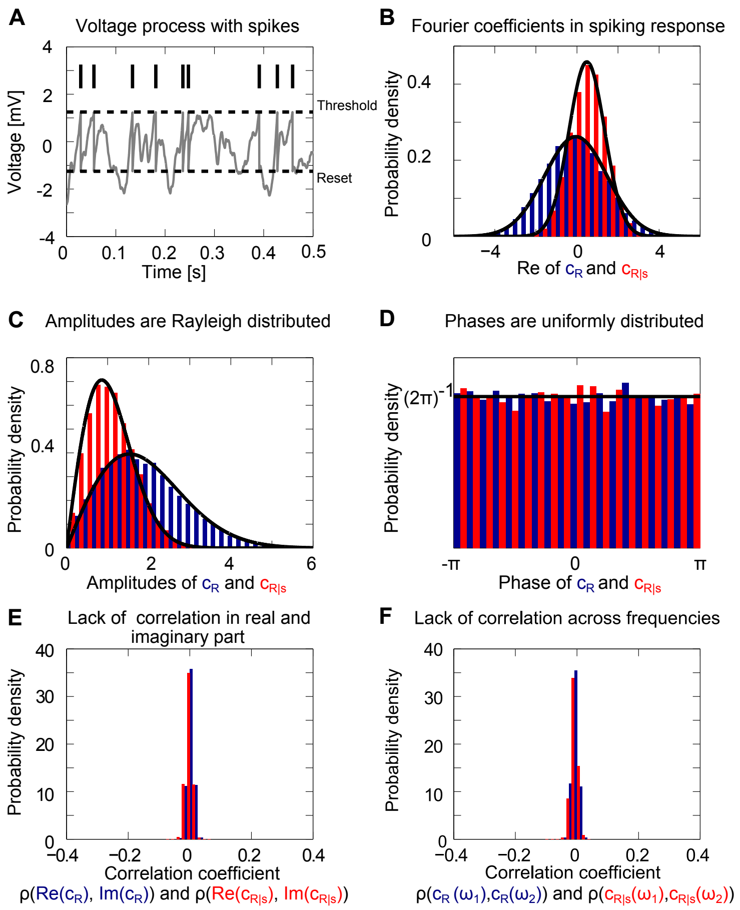

Supplementary Figure 3: Fourier statistics of exponential integrate-and-fire spiking responses (EIF) evoked by Ornstein-Uhlenbeck stimuli from Fig. 6 G in the main article. Voltage trajectory and spikes in a EIF spiking neuron. In this model a spike (black vertical line) is emitted whenever the voltage crosses a threshold value, after a spike voltage is reset to its reset value. Here, the firing rate is 25 Hz and the mean interspike interval is 40 ms. (**B**) Probability distribution of Fourier coefficients $c_R(\omega)$ (blue) and $c_{R|s}(\omega)$ (red) in spiking responses from (A). (C) We can confirm that the amplitudes are Rayleigh-distributed and that the phases are uniformly distributed (**D**). Black lines in (C) and (D) denote the respective fits. In (**E**) we numerically confirm that the real and imaginary part for each frequency between 1 and 500 Hz are indeed uncorrelated, as mathematical proofs indicate. (**F**) Similar lack of correlation holds across different frequencies ($\Delta f = 1$ Hz). We note that the deviation of the mean correlation coefficient in (E) and (F) is a finite size effect that can be further reduced by increasing the recording duration and trial number. In this figure we present Fourier statistics at $\omega = 8\pi$ Hz, $\tau_{mem} = 10$ ms, analogous statistics can be observed at other frequencies and membrane time constants.

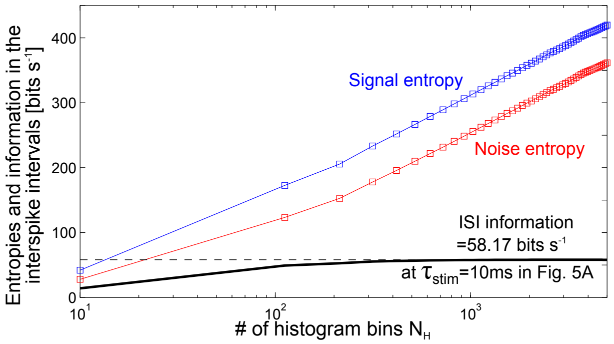

Supplementary Figure 4: Convergence of the ISI information as a function of discretisation. The signal and noise entropies (red and blue squares) calculated as described in the method section are proportional to the inverse of the number of bins N_H which were used to obtain the probability distributions $P(ISI)$ and $P(ISI|s)$ from the respective histograms. The resulting ISI information, the difference between signal and noise entropies, saturates close to $N_H = 1000$ for all points in Fig. 5 of the main manuscript. In this supplementary figure, we show data from Fig. 5 A evaluated at $\tau_{stim} = 10$ ms in the leaky integrate and fire neuron. For all values of the ISI information in Fig. 5 we display the information values which were we reached at saturation, $N_H \geq 1000$.

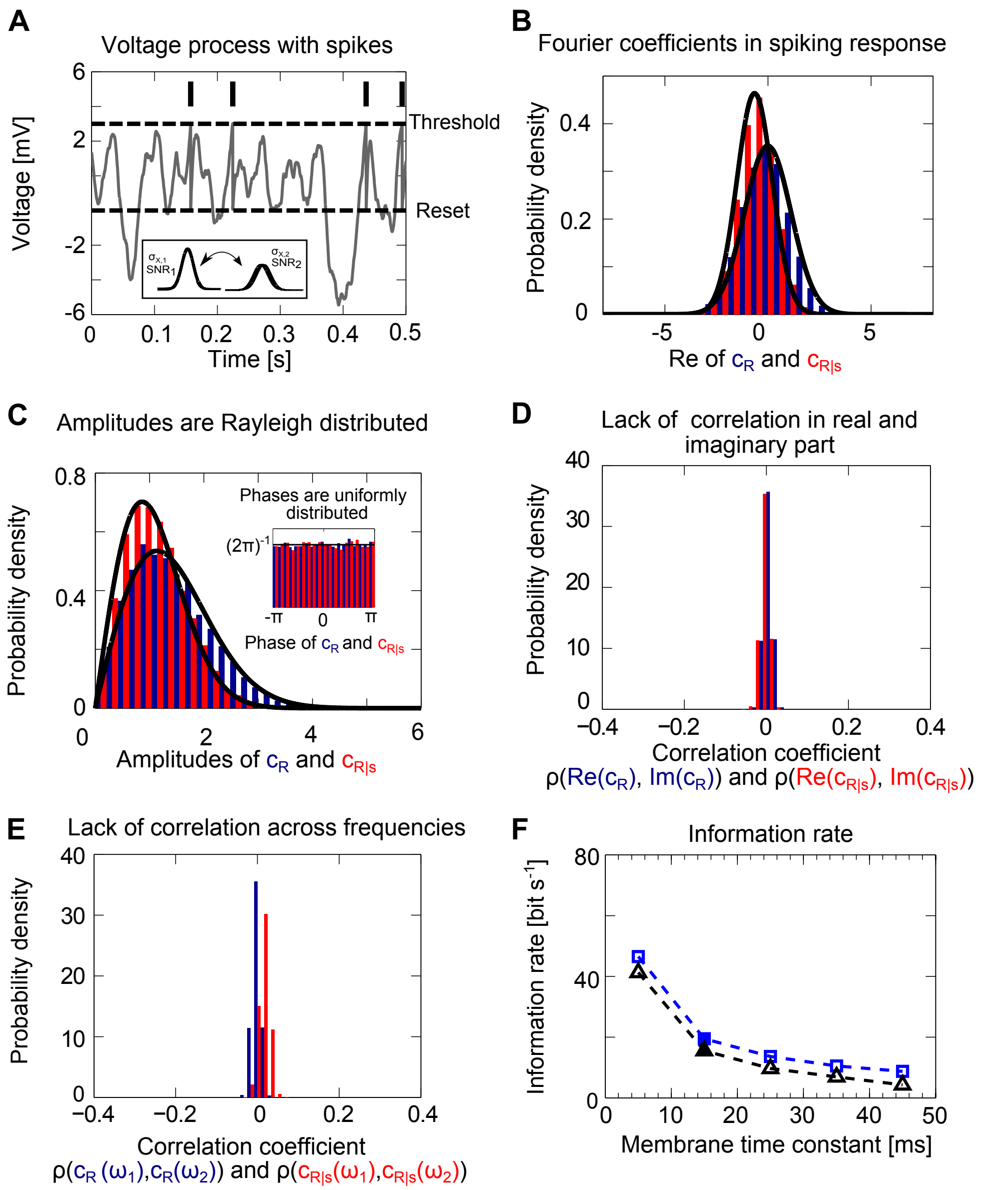

Supplementary Figure 5: Fourier statistics of leaky integrate-and-fire spike trains evoked by a stochastic switching process alternating between two Ornstein-Uhlenbeck states. (A) Voltage and spikes evoked by a stimulus alternating stochastically between two Ornstein-Uhlenbeck states. The two states differ in their variance and stimulus-to-noise ratios. Spikes are denoted by black vertical lines. (B) Probability distribution of Fourier coefficients $c_R(\omega)$ (blue) and $c_{R|s}(\omega)$ (red) from spiking responses from (A). (C) Amplitudes of Fourier coefficients c_R and mean normalized $c_{R|s}$ are Rayleigh distributed and their phases are uniform (C inset). Black lines denote the respective Rayleigh and uniform fits. In (B) and (C) we considered Fourier statistics at $\omega = 10\pi$ Hz. (D) Distribution of correlation coefficients between the real and the imaginary part for each frequency between 1 and 500 Hz, confirming the absence of correlation implied by mathematical proofs. (E) Similar lack of correlation in c_R and $c_{R|s}$ holds across different frequencies ($\Delta f = 1$ Hz). (F) Information content given by the correlation theory (blue squares) and the direct method (black triangles) yield equivalent results across two orders of magnitude in membrane time constants.

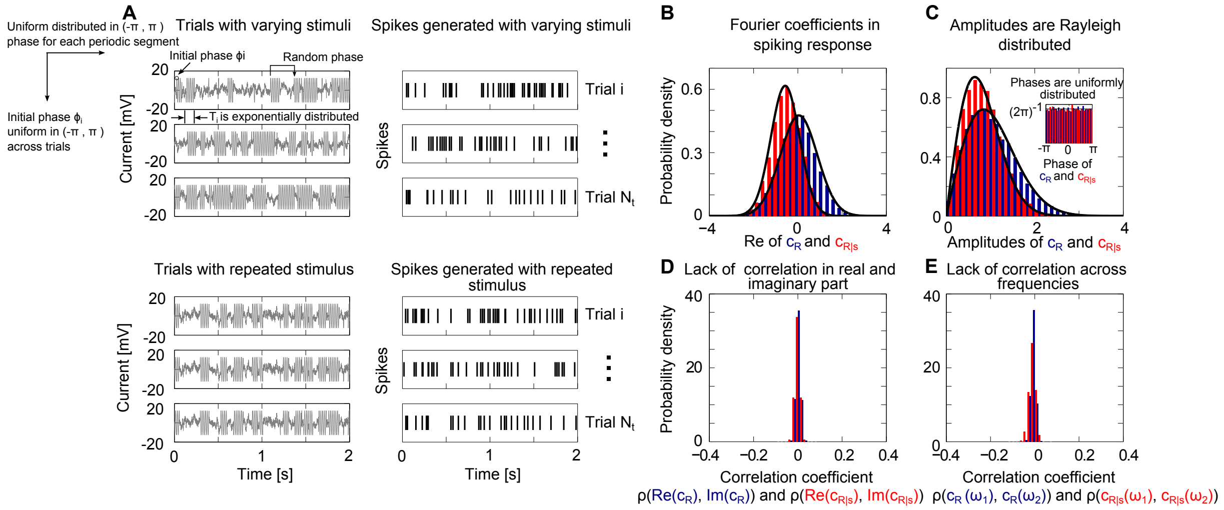

Supplementary Figure 6: Fourier statistics of leaky integrate-and-fire spike trains evoked by stationary switching processes. (A) Three trials with varying stimuli (left top) and repeating stimuli (left bottom). The resulting spike trains evoked by varying and repeating stimuli are shown (right top and right bottom). In this example, the stimuli and noise processes follow the same distribution and alternate stochastically (Poisson process, average switching rate 10 Hz) between an Ornstein-Uhlenbeck process and a sinusoid. At the beginning of the recording and at the onset of each sinusoidal segment the initial phase is reset to a random value $\phi_i \in [-\pi, \pi]$. (B) Probability distribution of Fourier coefficients $c_R(\omega)$ (blue) and $c_{R|s}(\omega)$ (red) in spiking responses in (A). (C) Amplitudes of Fourier coefficients c_R and mean normalized $c_{R|s}$ are Rayleigh distributed, their while the phases of c_R and mean normalized $c_{R|s}$ are uniform (C inset). Black lines denote the respective Gaussian and Rayleigh fits. (D and E) We confirm the predictions of the correlation theory that the real and imaginary part for each frequency between 1 and 500 Hz are uncorrelated in c_R and $c_{R|s}$. (E) Similar lack of correlation in c_R and $c_{R|s}$ holds across different frequencies ($\Delta f = 1Hz$). In (B)-(E) Fourier statistics for $\omega = 18\pi$ Hz is shown, parameter values of the leaky integrate and fire model are given on p. 6-7, membrane time constant $\tau_{mem} = 25$ ms, which corresponds to the leftmost point in Fig. 7.

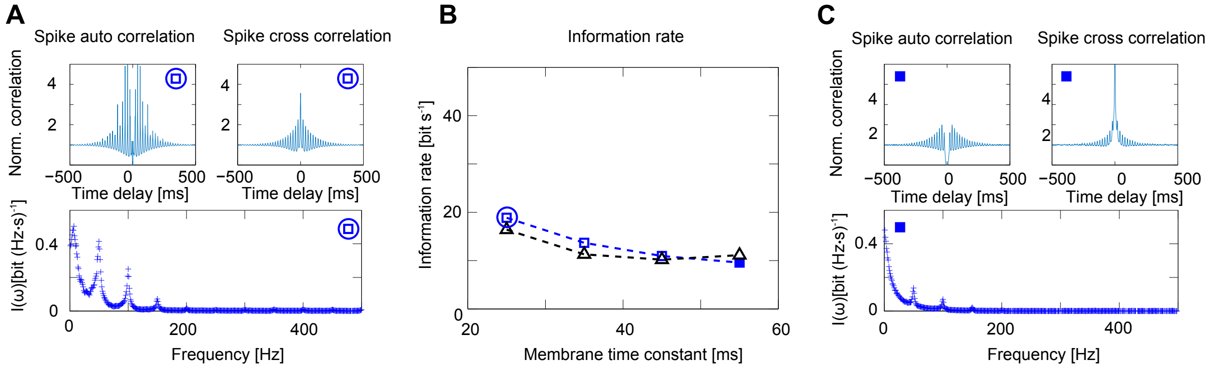

Supplementary Figure 7: Information content and spike correlation functions in leaky integrate-and-fire spike trains evoked by stationary switching processes from Fig. 6. (A, top) Spike auto and cross correlation functions at $\tau_{mem} = 25$ ms, each is normalized by the firing rate squared for better comparison. We note that both the auto and cross correlation functions decay to zero for long time delays. This indicates that spike trains have finite memory. (A, bottom) Information rate per frequency $I(\omega)$ as calculated from correlation functions in (A,top). (B) Information content given by the correlation theory (blue) and the direct method (black). As expected from finite memory and stationarity of the spikes the information content, the information content given by the direct method and correlation theory are in agreement. (C) Spike auto and cross correlation functions at $\tau_{mem} = 55$ ms, each is normalized by the firing rate squared for better comparison. We note that both the auto and cross correlation functions decay to zero for long time delays. This indicates that spike trains have finite memory. (C, bottom) Information rate per frequency $I(\omega)$ as calculated from correlation functions in (C,top)

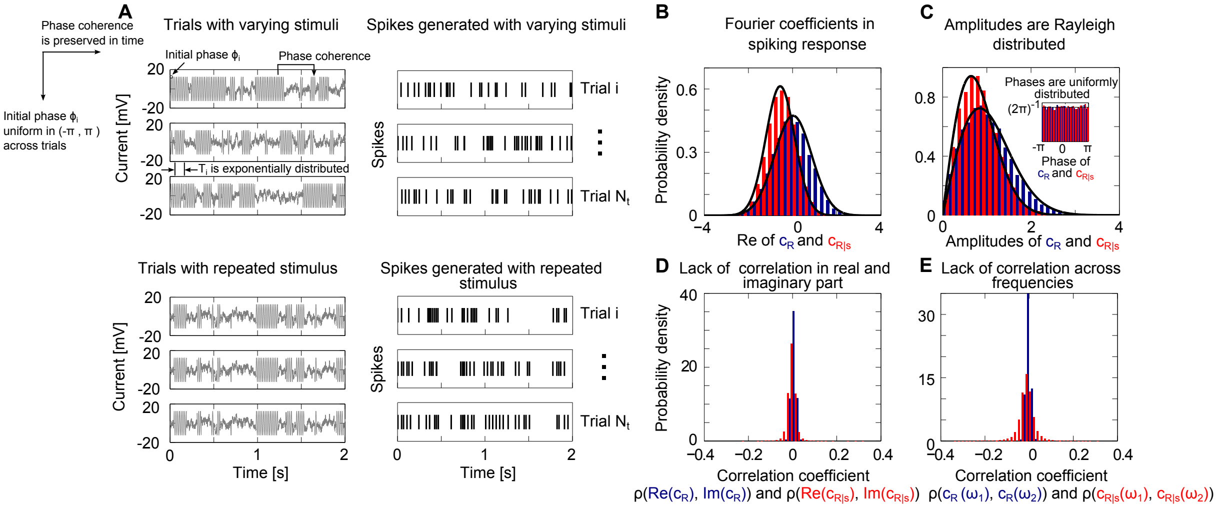

Supplementary Figure 8: Fourier statistics of leaky integrate-and-fire spike trains evoked by partially stationary switching processes. (A) Three trials with varying stimuli (left top) and repeating stimuli (left bottom). The resulting spike trains evoked by varying and repeating stimuli (right top and right bottom). Here, the stimuli and noise processes follow the same distribution and alternate stochastically (Poisson process, average switching rate 10 Hz) between an Ornstein-Uhlenbeck process and a sinusoid. At the beginning of the recording the phase is randomly selected but at each subsequent onset of a sinusoidal segment the phase is selected such that phase coherence to the last sinusoid segment is maintained. (B) Probability distribution of Fourier coefficients $c_R(\omega)$ (blue) and $c_{R|s}(\omega)$ (red) in spiking responses in (A). (C) Amplitudes of Fourier coefficients c_R and mean normalized $c_{R|s}$ are Rayleigh distributed, their while the phases of c_R and mean normalized $c_{R|s}$ are uniform (C inset). Black lines denote the respective Gaussian and Rayleigh fits. (D and E) Real and imaginary part for each frequency between 1 and 500 Hz are uncorrelated in c_R and $c_{R|s}$. (E) Correlation coefficients of c_R and $c_{R|s}$ across different frequencies ($\Delta f = 1Hz$) have a zero-centered distributions. The distribution of cross frequency correlation coefficients for $c_{R|s}$ has long tails suggesting cross frequency coupling. In (B)-(E) Fourier statistics for $\omega = 18\pi$ Hz is shown, parameter values of the leaky integrate and fire model are given on p. 6-7, membrane time constant $\tau_{mem} = 25$ ms, which corresponds to the leftmost point in Fig. 9.

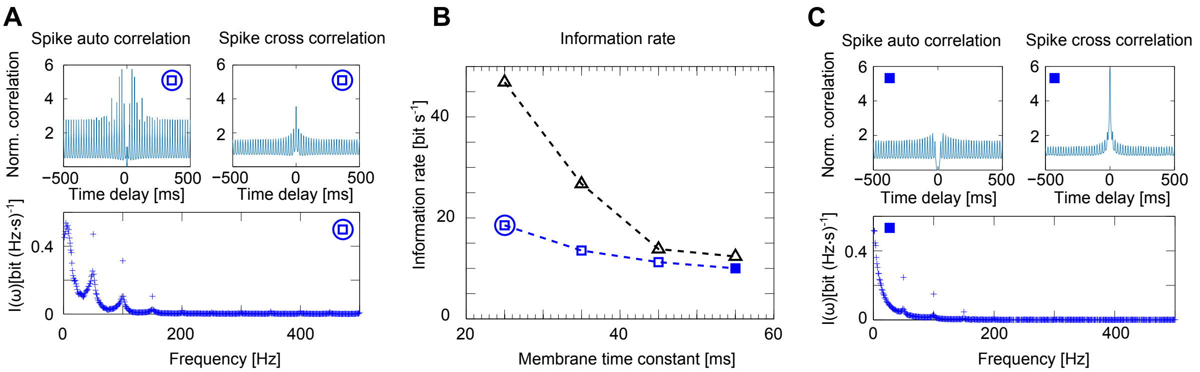

Supplementary Figure 9: Information content and spike correlation functions in leaky integrate-and-fire spike trains evoked by partially stationary switching processes from Fig. 8. (A, top) Spike auto and cross correlation functions at $\tau_{mem} = 25$ ms, each is normalized by the firing rate squared for better comparison. We note that neither the auto nor the cross correlation function decay to zero for long time delays. This indicates that spike trains have infinitely long phase coherence and therefore infinite memory. (A, bottom) Information rate per frequency $I(\omega)$ as calculated from correlation functions in (A). (B) Information content given by the correlation theory (blue) and the direct method (black). As expected from non-stationary spike trains with infinite memory neither direct method nor correlation theory are valid exactly and therefore result in different information values. While the information predicted by the correlation theory agrees with the finite memory contribution from Fig. 9 B, the direct method leads to comparable results only for longer membrane time constants. (C) Spike auto and cross correlation functions at $\tau_{mem} = 55$ ms, each is normalized by the firing rate squared for better comparison. We note that neither the auto nor the cross correlation function decay to zero for long time delays. This indicates that spike trains have infinitely long phase coherence and therefore infinite memory. (C, bottom) Information rate per frequency $I(\omega)$ as calculated from correlation functions in (C, top)

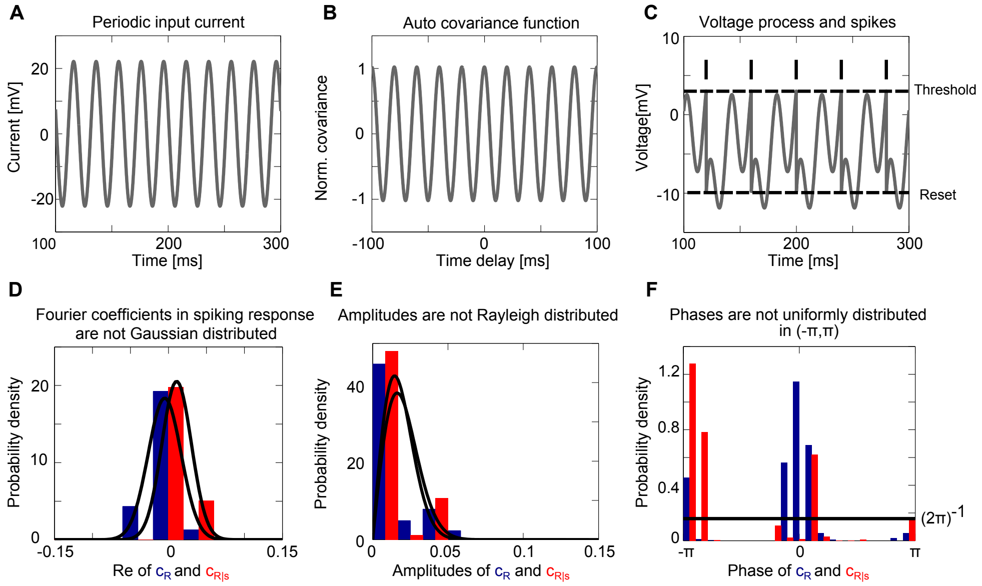

Supplementary Figure 10: Fourier statistics of leaky integrate-and-fire spike trains evoked by periodic inputs. (**A**) Trajectory of a periodic input (50 Hz). (**B**) Periodic auto covariance function of the stimulus shown in (A). (**C**) Periodic voltage trajectory and spikes (black vertical lines) of a LIF neuron evoked by the periodic input shown in (A). (**D**) Non-Gaussian probability distribution of Fourier coefficients $c_R(\omega)$ (blue) and $c_{R|s}(\omega)$ (red) in spiking responses evoked by stimuli drawn from (A). As expected from non-stationary spike trains with infinite memory, we can confirm that the amplitudes are not Rayleigh-distributed (**E**) and that the phase are not uniformly distributed (**F**). For reference black lines denote the Gaussian (D), Rayleigh (E) and uniform (F) distributions calculated using the measured variances. In D-F ω is equal to 8π Hz.

Table 1: Parameter sets and binary word statistics across neuron models in Fig. 6

**Threshold based neuron (Fig. 6A)**				
τ_{mem} [ms]	Firing rate [Hz]	$\langle ISI \rangle$ [ms]	Window sizes [ms] / bin size [ms]	Binary word stats 3. window size window/# bins/p(1 spike)/p(> 1 spikes)
1	45.1	21.7	{10,12,16,22,40}/2	16 ms/ 8 / 35.3% / 15.9%
3.2	24.8	38.98	{10,12,16,22,40}/2	16 ms / 8 / 30.2% / 4.4%
10	13.0	73.78	{10,12,16,22,40}/2	16 ms/ 8 / 18.6% / 1.1%
32	5.9	152.98	{10,12,16,22,40}/2	16 ms/ 8 / 8.8%/ 0.3%
100	1.7	406.66	{60,74,96,137,240}/12	96 ms/ 8 / 11.2% / 2.2%
**Leaky integrate-and-fire (Fig. 6E)**				
τ_{mem} [ms]	Firing rate [Hz]	$\langle ISI \rangle$ [ms]	Window sizes [ms] / bin size [ms]	Binary word stats 3. window size window/# bins/p(1 spike)/p(> 1 spikes)
3.2	107.5	8.7	{10,12,16,22,40}/2	16 ms / 8 / 15.2% / 36.9%
10	29.3	32.6	{10,12,16,22,40}/2	16 ms / 8 / 19.9% / 11.5%
32	6.7	138.2	{50,60,80,110,200}/10	80 ms / 8 / 28.8% / 11.1%
100	1.1	753.7	{50,60,80,110,200}/10	80 ms / 8 / 8.7% / 0.04%
**Adaptive leaky integrate-and-fire (Fig. 6F)**				
τ_{mem} [ms]	Firing rate [Hz]	$\langle ISI \rangle$ [ms]	Window sizes [ms] / bin size [ms]	Binary word stats 3. window size window/# bins/p(1 spike)/p(> 1 spikes)
3.2	200	4.9	{10,12,16,22,40}/2	16 ms / 8 / 12.6% / 42.1%
10	49.9	19.2	{10,12,16,22,40}/2	16 ms / 8 / 11.1% / 18.8%
32	8.6	104.3	{50,60,80,110,200}/10	80 ms / 8 / 14.7% / 16.9%
100	0.99	802.07	{50,60,80,110,200}/10	80 ms / 8 / 6.2% / 0.8%
**Adaptive leaky integrate-and-fire (Fig. 6F, inset)**				
τ_{ω} [ms]	Firing rate [Hz]	$\langle ISI \rangle$ [ms]	Window sizes [ms] / bin size [ms]	Binary word stats 3. window size window/# bins/p(1 spike)/p(> 1 spikes)
3.2	51.6	18.6	{25,30,40,55,100}/5	40 ms / 8 / 10.2% / 33.4%
5	49.9	19.2	{25,30,40,55,100}/5	40 ms / 8 / 13.8% / 34.9%
10	49.7	19	{25,30,40,55,100}/5	40 ms / 8 / 19.5% / 40%
31	51.4	20.1	{25,30,40,55,100}/5	40 ms / 8 / 22.6% / 46.6%
100	50.6	20.4	{50,60,80,110,200}/10	80 ms / 8 / 14.7% / 73.8%
316	50.6	20.4	{50,60,80,110,200}/10	80 ms / 8 / 14.5% / 70.9%
**Exponential integrate-and-fire (Fig. 6G)**				
τ_{mem} [ms]	Firing rate [Hz]	$\langle ISI \rangle$ [ms]	Window sizes [ms] / bin size [ms]	Binary word stats 3. window size window/# bins/p(1 spike)/p(> 1 spikes)
3.2	91.1	10.8	{80,96,128,176,320}/16	128 ms / 8 / 2.6% / 95.6%
10	24.5	40.3	{80,96,128,176,320}/16	128 ms / 8 / 16.7% / 70.9%
32	5.5	169.4	{160,192,256,352,640} / 32	256 ms / 8 / 33.7% / 41.3%
100	0.85	960.7	{160,192,256,352,640} / 32	256 ms / 8 / 20.3% / 0.84%

Supplementary Note 1
Additional mathematical details and derivations for correlation theory (equations 4 and 5)

Here, we provide additional mathematical details and derivations for the correlation theory of neural information. To help our readers with its numerical implementation we also provide computer code online [1].

We start by elaborating on the statistical properties we build on. We consider stationary spike trains $r(t) = \sum_j \delta(t - t_j)$ with finite memory and finite, non-zero coefficient of variation. Here, t_j are the spike times and $\delta(\cdot)$ is the Dirac delta distribution, see p. 9 in [2]. Similarly, the input current that drives a neuron's decision to spike consists of stimuli and noise processes which are independent of each other and each is stationary as a function of time and has finite temporal memory, finite means and finite variances. Note, that the spike generation mechanism in our theory is also time invariant, it preserves these quantities while it transforms inputs (a combination of stimuli and noise) into spike trains. Examples of such spike generation mechanisms include the four types of integrate-and-fire type neuron models which we described in the methods section of the main manuscript. Here, we now provide more details on the mathematical definitions of stationarity and finite memory.

Stationarity: A process $r(t)$ is stationary or "time invariant" if the joint distribution of $r(t_i), ..., r(t_n)$ is equal to the joint distribution of $r(t_{i+\tau}), ..., r(t_{n+\tau})$ for all τ. In other words,

$$\mathrm{P}(s(t_i), ..., s(t_n)) = \mathrm{P}(s(t_{i+\tau}), ..., s(t_{n+\tau})) \quad \text{for all } n \text{ and } \tau. \tag{1}$$

This means that once the probability distribution of the process is specified, it remains the same across time. In particular, this means that the mean and the auto covariance function are also time invariant

$$\mathrm{E}[s(t)] = \langle s(t) \rangle_{trials} = \langle s(t) \rangle_{time} = \mu_s \quad \text{for all } t, \tag{2}$$

$$\mathrm{Cov}[s(t), s(t+\tau)] = \mathrm{Cov}(\tau) \quad \text{for all } t. \tag{3}$$

This standard requirement of stationarity is necessary to define any probability density and calculate the information content. Should this requirement not be fulfilled by a given neural system, it is typically nevertheless possible to consider information content and probabilities, however, only for shorter segments of time, segments that are much smaller than the time scale of the system evolution.

Finite memory, finite coefficient of variation In a system with finite memory the interactions between any two time points vanish if the two points are sufficiently far apart [3,4]. In other words the values $r(t_i)$ and $r(t_j)$ are only correlated if $|t_j - t_i| < T_C$, where T_C is the finite correlation time of the process. Mathematically, it implies

$$\lim_{|\tau| \to \infty} \mathrm{Cov}(\tau) = 0. \tag{4}$$

Finite coefficient of variation is guaranteed if the mean and variance are both finite and non-zero, $0 < \langle r(t) \rangle_{time} < \infty$ and $0 < \langle r(t)^2 \rangle_{time} < \infty$, respectively. We will use these three properties later in the application of the Central Limit Theorem to the Fourier coefficients to show that they converge to a stable Gaussian distribution. Let us note that finite mean and finite variance can be achieved in time continuous as well as discrete point processes which may be infinite at some points (e.g. spike trains $\sum_{t_j} \delta(t - t_j)$).

We also note that the properties of finite memory and finite variability are plausible for any biological system where molecular constituents have finite lifetimes and finite operational ranges. Overall, we require rather weak statistical properties and do not specify any functional form of interactions. Processes fulfilling these stationarity and finite memory criteria may include Markovian, non-Markovian, Gaussian, non-Gaussian, time continuous or time discrete processes.

Fourier statistics of spike trains We consider a spike train $r(t) = \sum_j \delta(t - t_j)$ that was recorded for a time period T. Following the ideas in [3] we are interested in the Fourier representation of this spike

train. Its complex Fourier coefficients $c(\omega)$ are given by

$$c(\omega) = \frac{1}{T} \sum_{t_j} \exp(i\omega t_j) \tag{5}$$

where $\omega = 2\pi f$. For finite recording lengths, the frequency f is discrete $f = n/T$, where n is an integer, but it gradually becomes continuous as T grows. We recapitulate two important properties of the spike train and its coefficients $c(\omega)$. First, spike trains are real processes. Therefore, Fourier coefficients for ω and $-\omega$ are linearly dependent, $c(\omega) = c^*(-\omega)$. This implies that it is sufficient to consider only positive frequencies to obtain the complete information content. Second, we assume that spike trains are stationary processes. This implies that the information the spikes convey is independent of the time frame when they are recorded, e.g. time segments $[0, T]$ and $[\Delta, T + \Delta]$ carry the same information. This means that by shifting the time reference by any arbitrary time amount Δ we can induce any phase shift ϕ in the Fourier coefficients $c(\omega) \to c(\omega) \cdot \exp(i\phi)$ without affecting the information content. From this follows that the phase carries no information.
To calculate the mutual information, we need to obtain the distribution $P(c_R(\omega))$ of Fourier coefficients $c_R(\omega)$ from spikes recorded in trials with varying stimuli and the distribution $P(c_{R|s}(\omega))$ of coefficients $c_{R|s}(\omega)$ from spikes recorded in trials with repeated presentations of stimulus s.
To obtain distributions $P(c_R(\omega))$ and $P(c_{R|s}(\omega))$, we recall the finite memory property and assume that the recording time T is much longer than the correlation time of the process, $T \gg T_C$. The Fourier coefficients thus contain a sum of largely uncorrelated variables. Therefore, we can apply the Central Limit Theorem and conclude that $c_R(\omega)$ and $c_{R|s}(\omega)$ will both converge towards a Gaussian random value. In essence, the Central Limit Theorem states that no matter what the distribution of $\exp(i\omega t_j)$ is, as long as it has finite variance, their sum will always be a Gaussian random variable. To make this more accessible for our readers we now highlight the Gaussianity of Fourier coefficients in Fig. 2 in the main manuscript. We now mathematically formalize this Gaussian intuition and use the proofs by Kawata [5] and Brillinger (Theorem 4.4.1 in [3] and [4], [6]) to derive $P(c_R(\omega))$ and $P(c_{R|s}(\omega))$ and their properties.

Obtaining the probability distribution $P(c_R(\omega))$ To this end, we consider trials with varying stimuli where at each trial stimuli and noise are drawn independently from their respective distributions such that the spike trains are also independent across trials. Subsequently, we investigate the distribution of spiking Fourier coefficients across trials. Brillinger and Kawaka have shown in their work that the distribution of Fourier coefficients $c_R(\omega)$ across trials follows a zero-mean, complex normal distribution with variance $\sigma_R^2(\omega)$ in the limit of infinitely long recording times T in trials with varying stimuli, such that

$$\mathrm{P}(c_R(\omega)) = \mathrm{P}\left(\frac{1}{T} \sum_j \exp(i\omega t_j)\right) \xrightarrow{T\to\infty} \mathcal{N}_c(0, \sigma_R^2(\omega)). \tag{6}$$

Furthermore, the distributions $P(c_R(\omega_1))$ and $P(c_R(\omega_2))$ for $\omega_1 \neq \omega_2$ are asymptotically independent for large T. In a complex normal distribution $\mathcal{N}_c(0, \sigma_R^2(\omega))$ the real and imaginary parts are statistically independent and Gaussian distributed with equal variance $\sigma_R^2(\omega)/2$ (see [3], [4]). Considering the fact that the Fourier coefficients $c_R(\omega)$ originate from a stationary process we also know that the amplitude of $c_R(\omega)$ but not its phase carries information about the stimulus structure. For completeness, let us note that in any given recording the spike times are typically discretized and can be numbered from 0 to $N \cdot dt$, where N is the last recording point and dt is the bin size. In this case a small, countable number of frequencies ($\omega = 0, \pm\pi/dt, \pm 2\pi/dt, ...$) leads to a real rather than complex Gaussian distribution. For example $\omega = 0$ and $\omega = \pi/dt$ lead to $c(0) = c(\pi/dt) = \frac{1}{T}\sum_{t_j} 1 = \nu$, where ν is the firing rate. Since, the Riemann integral over the frequency space is not affected by this small, countable set of frequencies [7] we do not consider this set in our calculations. In the continuous limit where $dt \to 0$ and $N \to \infty$ the only ω-exception is $\omega = 0$, a sole value which does not alter the value of Riemann integral over the Fourier frequencies and which we therefore leave out in our further calculations.

Obtaining the probability distribution $P(c_{R|s}(\omega))$ To this end, we consider trials with repeating

stimuli where at each trial the noise is drawn independently from its distribution but the temporal stimulus trajectory repeats itself. This results in spike trains, which are correlated across trials. Here, we investigate the distribution of spiking Fourier coefficients across trials. Following the arguments of Brillinger and Kawaka, and using the assumption that stimulus and noise processes are independent of each other and each is selected from a set of temporally stationary, finite memory processes, we find that this leads again to the distribution of Fourier coefficients across trials is a complex Gaussian distribution $\mathrm{P}(c_{R|s}(\omega))$ which is described by

$$\mathrm{P}(c_{R|s}(\omega)) = \mathrm{P}\left(\frac{1}{T}\sum_j \exp(i\omega t_j)\right) \xrightarrow{T\to\infty} \mathcal{N}_c(\mu, \sigma^2_{R|s}(\omega)). \tag{7}$$

In repeated presentations of the same stimulus which we consider here, the Fourier coefficients $c_{R|s}(\omega)$ will have a finite, non-zero mean μ which is determined by the specific trajectory of the chosen stimulus. But since the trajectory of the stimulus and noise are independent and the recording length T is longer than the time scale of both temporal interactions, the chosen temporal stimulus trajectory will not influence the noise trajectory and its variance. While the stimulus remains the same across these trials, the noise varies across trials and is independent from trial to trial. Furthermore, the spike mechanism itself is time invariant such that the spikes inherit the finite memory property for any combination of stimulus and noise processes and their temporal statistics are independent of the start and end point of the recording. This leads to two observations. First, due to invariance of stimulus statistics with respect to the start and end point of the recording the amplitude of the Fourier coefficients $c_{R|s}(\omega)$ but not their phase carries information about the stimulus structure. Second, to calculate the distribution of $c_{R|s}(\omega)$ the Central Limit Theorem can be applied in the same manner as for the coefficients $c_R(\omega)$. This situation is illustrated in Fig. 2 in the main manuscript and Figs 1, 2 and 3 where both the complex Gaussianity and independence of frequencies is confirmed numerically.
To mathematically support this argument further we refer to p. 38 Theorem 2.9.1 [3]. This theorem shows that the output of a time invariant spiking mechanism that receives stationary inputs with finite memory is also a process which is stationary and has finite memory and thus has complex Gaussian Fourier coefficients that are independent across frequencies. We follow the arguments by Brillinger and consider a Volterra functional expansion which is given by

$$r(t) = \sum_{j=0}^{L} \sum_{u_1,\ldots,u_j} a_j(t-u_1,\ldots,t-h_j)X(u_1)\cdot\ldots\cdot X(u_j), \tag{8}$$

where $X(u_i)$ is the input at time u_i and $a_j(t)$ are system specific Volterra kernels. The input is a function of the stimulus and noise $X(u_i) = f(s(u_i), n(u_i))$, which can be either a simple addition or any other more complex relation. Since the spiking output is a sum of input processes weighted by the Volterra kernels, its finite memory properties and invariance with respect to time shifts are directly inherited from the inputs. Additionally, we recognize that the terms proportional to the stimulus (e.g. $a_j(t-u_1)s(u_1)$) will contribute to the finite mean μ of the complex Gaussian Fourier distribution $\mathrm{P}(c_{R|s}(\omega))$ which is obtained across trials with repeated stimulus presentations. On the other hand, the terms proportional to the noise process (e.g. $a_j(t-u_1)n(u_1)$ or $a_j(t-u_1)s(n_j)n(u_1)$) will lead in the Fourier space to a Gaussian distribution across trials and will contribute to the variance $\sigma^2_{R|s}(\omega)$ of $\mathcal{N}_c(\mu, \sigma^2_{R|s}(\omega))$. In other words, the common stimulus component contributes to the mean Fourier coefficient while the noise is responsible for the Gaussian distribution. Importantly, the noise-induced variability across trials, but not the mean, determine the correlations across frequencies. Because the noise process has the same statistical properties of stationarity and finite memory, as the combination of stimulus and noise in trials with varying stimuli, the same arguments that lead to the independence of frequencies for $\mathrm{P}(c_R(\omega))$ in the proof by Brillinger apply here again. We thus obtain the result in equation (7) along with the statement that the distributions $P(c_R(\omega_1))$ and $P(c_R(\omega_2))$ for $\omega_1 \neq \omega_2$ are asymptotically independent for large T. We numerically confirm these mathematical arguments in Fig. 2 of the main manuscript for bimodal inputs in the leaky integrate-and-fire model and in Figs. 1-3 of this supplementary material for three types of leaky integrate-and-fire models and in Figs. 5 and 6 for stochastic switching processes.

Details and derivations of mutual information Here, we consider the mutual information for each

frequency component as defined by

$$I(\omega) = H_{signal}(\omega) - H_{noise}(\omega) = -\sum_R P(c_R(\omega)) \log_2 P(c_R(\omega)) + \left\langle \sum_R P(c_{R|s}(\omega)) \log_2 P(c_{R|s}(\omega)) \right\rangle_s \tag{9}$$

where $\langle\rangle_s$ denotes the average over all possible stimuli. Dealing with the complex Gaussian distributions $P(c_{R|s}(\omega))$ and $P(c_R(\omega))$ which have additional symmetries with respect to phase shifts, we obtain the following result

$$I(\omega) = \frac{1}{2} \log_2 \left(\sigma_R^2(\omega) / \sigma_{R|s}^2(\omega) \right). \tag{10}$$

To derive this result, we consider two important statements. First, the differential mutual information of a (complex) Gaussian variable does not depend on its mean value, a property typically referred to as "translational invariance" (pp.250 and 253 [8]). Second, the phases of the complex Gaussian Fourier coefficients $c_R(\omega)$ and $c_{R|s}(\omega)$ carry no additional information because the statistics of spiking process are independent of the start and end point of the recording, all information is contained already in the amplitude of these variables. Taking these statements as a starting point we can write

$$I(\omega) = H^A_{signal} - H^A_{noise}, \tag{11}$$

where the superscript A denotes the Fourier amplitude for which the signal and noise entropies are calculated. Using the property of translational invariance, we can zero center the distribution of $c_{R|s}(\omega)$. We are now dealing with zero mean complex Gaussian coefficients $c_{R|s}(\omega)$ and $c_R(\omega)$ whose amplitudes can now be described by a Rayleigh distribution [9]. Importantly, the variance of the complex Gaussian distribution is equal to the variance of the Rayleigh distribution. Now, we can use the entropy of a Rayleigh distributed variable with variance σ^2 which is given by $H = \frac{1}{2}\log_2(\frac{1}{2}\sigma^2 e^{2+\Gamma})$, where Γ is Euler-Mascheroni constant [10], to calculate the mutual information rate per frequency for our quantities of interest. We obtain

$$I(\omega) = H^A_{signal}(\omega) - H^A_{noise}(\omega) \tag{12}$$

$$= \frac{1}{2}\log_2(\frac{1}{2}\sigma_R^2(\omega)e^{2+\Gamma}) - \frac{1}{2}\log_2(\frac{1}{2}\sigma_{R|s}^2(\omega)e^{2+\Gamma}) \tag{13}$$

$$= \frac{1}{2}\log_2\left(\frac{\frac{1}{2}\sigma_R^2(\omega)e^{2+\Gamma}}{\frac{1}{2}\sigma_{R|s}^2(\omega)e^{2+\Gamma}}\right) = \frac{1}{2}\log_2\left(\sigma_R^2(\omega)/\sigma_{R|s}^2(\omega)\right). \tag{14}$$

We thus obtain the result in equation (10). We note that a division by a $\sigma_{R|s}^2(\omega)$ is possible because this quantity is non-zero. This is due to residual variability remaining in the response across trials due to noise, even in trials with repeated stimulus s presentations. Following the results of Brillinger [3] which we summarized above we also know that each frequency ω contributes independently to mutual information. We thus can sum their contributions and obtain

$$I(R,S) = \frac{1}{2}\int_0^\infty \log_2\left(\sigma_R^2(\omega)/\sigma_{R|s}^2(\omega)\right) d\omega. \tag{15}$$

Note that because the spike trains are real processes the Fourier coefficients for ω and $-\omega$ are linearly dependent and the contribution of positive frequencies is sufficient for the calculation of information. We now proceed to calculate the variances $\sigma_R^2(\omega)$ and $\sigma_{R|s}^2(\omega)$.

Details on the variance calculations for $\boldsymbol{\sigma_R^2(\omega)}$ and $\boldsymbol{\sigma_{R|s}^2(\omega)}$ Here we show that variances $\sigma_R^2(\omega)$, $\sigma_{R|s}^2(\omega)$ can be identified with the spike auto and cross correlation functions. We start by defining the variance of a coefficient $c(\omega)$ and obtain

$$\sigma^2(\omega) = \langle c(\omega)c(\omega)^*\rangle_{trial} - \langle c(\omega)\rangle_{trial}\langle c(\omega)^*\rangle_{trial}. \tag{16}$$

where $\langle\cdot\rangle_{trial}$ denotes the average over the statistical ensemble and * the complex conjugate. For a given set of N_T trials, we now calculate $\sigma_R^2(\omega)$ and $\sigma_{R|s}^2(\omega)$ of the Fourier coefficients $c_R(\omega)$ and $c_{R|s}(\omega)$. We next evaluate

$$\sigma^2(\omega) = \left[\sum_n^{N_T} c(\omega)_{trial_n} c(\omega)^*_{trial_n} - (\sum_n^{N_T} c(\omega)_{trial_n})(\sum_m^{N_T} c(\omega)^*_{trial_m})/N_T\right]/(N_T-1) \tag{17}$$

$$= \left[\sum_n^{N_T} c(\omega)_{trial_n} c(\omega)^*_{trial_n} - \sum_n^{N_T} c(\omega)_{trial_n} c(\omega)^*_{trial_n}/N_T - (\sum_n^{N_T}\sum_{n\neq m}^{N_T} c(\omega)_{trial_n} c(\omega)^*_{trial_m})/N_T\right]/(N_T-1), \tag{18}$$

thus for large N_T we obtain

$$\sigma^2(\omega) = \left\langle c(\omega)_{trial_n} c(\omega)^*_{trial_n}\right\rangle_{trial} - \left\langle c(\omega)_{trial_n} c(\omega)^*_{trial_m}\right\rangle_{trial_n\neq trial_m}. \tag{19}$$

For Fourier coefficients $\sigma_R^2(\omega)$ and $\sigma_{R|s}^2(\omega)$ we obtain

$$\sigma_R^2(\omega) = \left\langle c(\omega)_{trial_n} c(\omega)^*_{trial_n}\right\rangle_{trial}, \tag{20}$$

$$\sigma_{R|s}^2(\omega) = \left\langle c(\omega)_{trial_n} c(\omega)^*_{trial_n}\right\rangle_{trial} - \left\langle c(\omega)_{trial_n} c(\omega)^*_{trial_m}\right\rangle_{trial_n\neq trial_m}. \tag{21}$$

Note, that the second term describing the cross correlations across different trials vanishes for $\sigma_R^2(\omega)$ but is finite in $\sigma_{R|s}^2(\omega)$. We now express these quantities via spike correlation functions. First we define the Fourier transform ($\mathcal{F}$) of the auto correlation function

$$C_{auto}^{spike}(\omega) = \mathcal{F}\left(C_{auto}^{spike}(\tau)\right) = \mathcal{F}\left(\frac{1}{T}\int_0^T r(t)r(t-\tau)\mathrm{d}t\right) \tag{22}$$

and yield the variance $\sigma_R^2(\omega)$ for presentations of varying stimuli

$$\sigma_R^2(\omega) = \frac{1}{T^2}\left\langle \sum_i^{N_S}\exp(i\omega t_i)\sum_j^{N_S}\exp(-i\omega t_j)\right\rangle_{trial} \tag{23}$$

$$= \frac{1}{T} r(\omega)r^*(\omega), \tag{24}$$

$$= \frac{1}{T} C_{auto}^{spike}(\omega), \tag{25}$$

where $r(\omega)$ is the Fourier transform of a spike train and N_S is the number of spikes. Second, we define the Fourier transform of the cross correlation function for trials $n \neq m$

$$C_{cross}^{spike}(\omega) = \mathcal{F}\left(C_{cross}^{spike}(\tau)\right) = \mathcal{F}\left(\frac{1}{T}\int_0^T r_{trial_n}(t) r_{trial_m}(t-\tau)\mathrm{d}t\right) \tag{26}$$

and with equivalent calculations obtain the variance $\sigma_{R|s}^2(\omega)$ for repeated stimulus presentation

$$\sigma_{R|s}^2(\omega) = \frac{1}{T}\left(C_{auto}^{spike}(\omega) - C_{cross}^{spike}(\omega)\right). \tag{27}$$

Inserting this in equation (14) it follows that the information rate per frequency is given by

$$I(\omega) = \frac{1}{2}\log_2\left(\frac{\sigma_R^2(\omega)}{\sigma_{R|s}^2(\omega)}\right) = \frac{1}{2}\log_2\left(\frac{\frac{1}{T}C_{auto}^{spike}(\omega)}{\frac{1}{T}\left(C_{auto}^{spike}(\omega) - C_{cross}^{spike}(\omega)\right)}\right) \tag{28}$$

$$= -\frac{1}{2}\log_2\left(1 - \frac{C_{cross}^{spike}(\omega)}{C_{auto}^{spike}(\omega)}\right), \tag{29}$$

and the information rate by

$$I(R,S) = -\frac{1}{2}\int_{-\infty}^{\infty} \log_2\left(1 - \frac{C_{cross}^{spike}(\omega)}{C_{auto}^{spike}(\omega)}\right) d\omega. \tag{30}$$

In Fig. 2 of the main manuscript we numerically confirmed that the Fourier coefficients $c_R(\omega)$ and $c_{R|s}(\omega)$ indeed show the mathematically predicted properties such as Gaussianity and independence of the real and imaginary part (Fig. 2 D,F), Rayleigh distribution of amplitudes and equal distribution of phases (Fig.2 E,G) in the leaky integrate-and-fire model neuron driven by bimodal stimuli.

Additional details on the derivation of the linear approximation of the full information content

One of the now classic linear approximations to the information content of a neuron has been proposed by Stein and colleagues in 1970s [11–14]. Here, we show that a linear approximation, also referred to as the *lower bound estimate* $I_{LB}(R,S)$, can be derived from our expression for the full information content (equation (30), or main manuscript equation (4)).
We linearize the nominator within the logarithm of equation (30), which is the spike cross correlation function $C_{cross}^{spike}(\omega)$. We linearly approximate the spiking response to an input stimulus $X(t)$ using the first Wiener kernel $\upsilon_1(\tau)$:

$$r(t) \approx r_{est}(t) = \int_{-\infty}^{\infty} \upsilon_1(\tau)X(t-\tau)\mathrm{d}\tau. \tag{31}$$

Here, the first Wiener kernel $\upsilon_1(\tau)$ is derived using the standard reverse correlation method [15]

$$\upsilon_1(\omega) = \frac{r(\omega)X^*(\omega)}{X(\omega)X^*(\omega)}. \tag{32}$$

Decomposing the input current $X(t)$ into the uncorrelated signal and noise part, $X(t) = s(t) + n(t)$, we obtain the linear approximation to the spike correlation function

$$C_{cross}^{spike}(\omega) \approx \langle \upsilon_1(\omega)s(\omega)\upsilon_1^*(\omega)s^*(\omega)\rangle. \tag{33}$$

Equipped with this result, we can now obtain the linear approximation to the information content:

$$I_{LB}(R,S) = -\frac{1}{2}\int_0^{\infty} \log_2\left(1 - \frac{\langle \upsilon_1(\omega)s^*(\omega)\upsilon_1^*(\omega)s(\omega)\rangle}{\langle r^*(\omega)r(\omega)\rangle}\right) \mathrm{d}\omega, \tag{34}$$

considering equation (32) we obtain

$$I_{LB}(R,S) = -\frac{1}{2}\int_0^{\infty} \log_2\left(1 - \frac{\langle s^*(\omega)r(\omega)\rangle\,\langle s(\omega)r^*(\omega)\rangle}{\langle s^*(\omega)s(\omega)\rangle\,\langle r^*(\omega)r(\omega)\rangle}\right) \mathrm{d}\omega \tag{35}$$

$$= -\frac{1}{2}\int_0^{\infty} \log_2\left(1-\gamma^2\right) \mathrm{d}\omega, \tag{36}$$

where the coherence function is denoted by $\gamma^2 = \frac{\langle s^*(\omega)r(\omega)\rangle\langle s(\omega)r^*(\omega)\rangle}{\langle s^*(\omega)s(\omega)\rangle\langle r^*(\omega)r(\omega)\rangle}$, see [13, 16]. This shows that the linear approximation of our general result recovers the form of the coherence based, seminal lower bound estimate of Stein and colleagues. Let us also note that the lower bound estimate is typically derived assuming Gaussian stimulus and response statistics [11–14]. Here, however we obtained this result by applying a linear approximation to our general result which holds for both Gaussian and non-Gaussian stimuli. Additionally, let us mention that the coherence function in equation (36) can be related to the signal-to-noise ratio $SNR(\omega)$ [12, 13]

$$I_{LB}(R,S) = \frac{1}{2}\int_0^{\infty} \log_2\left(1 + SNR_{LB}(\omega)\right) \mathrm{d}\omega, \tag{37}$$

where $SNR_{LB}(\omega) = \frac{\langle s_{est}^*(\omega)s_{est}(\omega)\rangle}{\langle n^*(\omega)n(\omega)\rangle}$ is the signal-to-noise ratio of the estimated signal $s_{est}(\omega)$ to the noise $n(\omega) = s(\omega) - s_{est}(\omega)$. The coherence function and the signal-to-noise ratio are related via $\frac{\gamma^2}{1-\gamma^2} = \frac{\langle s_{est}^*(\omega)s_{est}(\omega)\rangle}{\langle n^*(\omega)n(\omega)\rangle}$.

Additional mathematical details on the derivation of the peristimulus time histogram auto correlation

In equation (15) of our manuscript, we used the identify relation between the auto correlation of the peristimulus time histogram (PSTH) and the spike cross correlation function to estimate the information contained in the stimulus-induced rate variations. Here, we derive this relation.
We start by defining the PSTH as the average firing rate across trials which was evoked by a repeating stimulus, $\mathrm{PSTH}(t) = r_s(t) = \frac{1}{N_T}\sum_i^{N_T} r_i(t)$. Here, N_T is the number of trials and $r_i(t)$ the firing rate at time t in trial i. The auto correlation function of the PSTH C_{auto}^{PSTH} is given by

$$C_{auto}^{PSTH}(\tau) = \langle r_s(t) \cdot r_s(t+\tau) \rangle_{time} \tag{38}$$

where $\langle\cdot\rangle_{time}$ denotes an average across time. Using the definition of the PSTH we obtain

$$\begin{aligned} C_{auto}^{PSTH}(\tau) &= \langle r_s(t) \cdot r_s(t+\tau) \rangle_{time} & (39) \\ &= \left\langle \frac{1}{N_T}\sum_i^{N_T} r_i(t) \cdot \frac{1}{N_T}\sum_j^{N_T} r_j(t+\tau) \right\rangle_{time} & (40) \\ &= \frac{1}{{N_T}^2} \left\langle \sum_i^{N_T} r_i(t) r_i(t+\tau) + \sum_i^{N_T}\sum_{i\neq j}^{N_T} r_i(t) r_j(t+\tau) \right\rangle_{time} & (41) \end{aligned}$$

Because the first term in equation (41) is of order N_T and the second of order N_T^2, only the second term will remain in the limit $N_T \to \infty$. We thus find

$$\begin{aligned} C_{auto}^{PSTH}(\tau) &= \lim_{N_T\to\infty} \frac{1}{{N_T}^2}\frac{1}{T}\int_0^T \left(\sum_i^{N_T} r_i(t) r_i(t+\tau) + \sum_i^{N_T}\sum_{j\neq i}^{N_T} r_i(t) r_j(t+\tau) \right) \mathrm{dt} & (42) \\ &= \frac{1}{{N_T}^2} \sum_i^{N_T}\sum_{j\neq i}^{N_T} \frac{1}{T}\int_0^T r_i(t) r_j(t+\tau)\mathrm{dt} = C_{cross}^{spike}(\tau) & (43) \end{aligned}$$

Finally, we apply apply the Fourier transformation and summarize the relation between the PSTH and the spike cross correlation

$$C_{cross}^{spike}(\omega) = C_{auto}^{PSTH}(\omega) = \mathrm{PSTH}(\omega) \cdot \mathrm{PSTH}^*(\omega). \tag{44}$$

Supplementary Note 2

Measuring neural information content

Here, we present four distinct ways to access neural information content from available spike data. The first method (see paragraph "Numerical implementation of correlation theory") is a numerical implementation of our correlation theory and relies on the evaluation of spike auto and cross correlation functions, see equation 5. The second method (see paragraph "Numerical implementation of the direct method") is the standard direct method proposed by [17], which relies on the probabilities evaluation of binary words. The third method (see paragraph "Information contained in the interspike interval distribution") evaluates the information contained in the interspike probabilities. This method measures only a part of the complete information content, the part encoded in the independent interspike intervals. In the main manuscript, Fig.6 presents the results of the first and second methods along with their accuracies. Fig.5 contrasts the approximate results of the third method with the exact solution provided by the first method and the correlation theory.

Numerical implementation of correlation theory

Here, we present a numerical algorithm to evaluate the spike auto and cross correlation functions in a data set. We consider a data set consisting of N_T trials recorded for varying stimuli and N_T trials

recorded while the same stimulus was presented and the noise varied. Each of the trials has a duration of T and a discretization time step of dt. The auto correlation function $C^{spike}_{auto}(\tau)$ is calculated from the trials with varying stimuli and the spike cross correlation function $C^{spike}_{cross}(\tau)$ is calculated from trials with a repeated stimulus presentation and averaged across $\#S$ different stimuli.
To calculate the spike cross correlation function $C^{spike}_{cross}(\tau)$ we have at our disposal $\frac{1}{2} \cdot N_T\,(N_T-1)$ possible pairs that can be drawn from the N_T trials, all or a subset of which can be evaluated. To make sure that auto and cross correlation functions are estimated with approximately the same precision we used N_T rather than all $\frac{1}{2} \cdot N_T\,(N_T-1)$ pairs. To increase precision this number can be customized, particularly in situations with a high trial-to-trial variability. To calculate the spike cross correlation function in pairs of trials we apply a Gaussian filter with a time width of σ_G to each spike and consider the contribution of spikes in the second spike train at time τ after each spike in the first spike train. For the spike auto correlation function we repeat this procedure considering the contribution of spikes within the same spike train. After obtaining both correlation functions $C^{spike}_{cross}(\tau)$ and $C^{spike}_{auto}(\tau)$ for time delay $\tau \in [-\tau_{max}, \tau_{max}]$ and discretization step δ_τ we verify the presence of two important features. First, both functions decay to zero for large τ such that the spike processes fulfill the finite memory condition. Second, the temporal structure of these functions is well-resolved and fully contained in the considered interval $\tau \in [-\tau_{max}, \tau_{max}]$. When necessary τ_{max} or δ_τ is modified to meet these requirements. In the subthreshold, fluctuation driven regime the neurons often have correlation times of a few tens of ms [18] such that $\tau_{max} \approx 200-400$ ms is sufficient to capture the full temporal correlation structure. In situations with oscillatory cross correlations, e.g. in the superthreshold firing regime, temporal correlations can extend over many hundreds of ms and therefore necessitate longer τ_{max} values.
The next step is a transformation of auto and cross correlation functions into the Fourier domain and the integration of their ratio according to equation 5 from zero to f_{max}.
For Fig.3 we considered the following parameters $\#S = 12$, $N_T = 5000$, $T = 40\,\mathrm{s}$, $dt = 0.015\,\mathrm{ms}$, $\tau_{max} = 200\,\mathrm{ms}$, $\sigma_G = 0.1\,\mathrm{ms}$ and $f_{max} = 500\,\mathrm{Hz}$. In Fig. 5 we set $\#S = 32$, $N_T = 1000$, $T = 10\,\mathrm{s}$, $dt = 0.05(0.01)\,\mathrm{ms}$, $\tau_{max} = 500\,\mathrm{ms}$, $\delta_\tau = 1(0.1)\,\mathrm{ms}$, $\sigma_G = 0.1(0.01)\,\mathrm{ms}$ and $f_{max} = 500(100)\,\mathrm{Hz}$. Values in brackets denote the parameters for $\tau_{stim} = 0.1-1$ ms in Fig. 5 A, which were particularly small and required higher temporal precision. In Fig. 6 we set $\#S = 32$, $N_T = 1000$, $T = 50\,\mathrm{s}$, $dt = 0.05\,\mathrm{ms}$, $\tau_{max} = 200\,\mathrm{ms}$, $\sigma_G = 0.1\,\mathrm{ms}$ and $f_{max} = 500\,\mathrm{Hz}$. For the exponential integrate-and-fire model in Fig. 6G we selected $\tau_{max} = 500\,\mathrm{ms}$ to fully capture its broader correlation functions.
To numerically evaluate the Fourier statistics in Fig. 2 and the Supplementary Figs. 1-3, we calculate in each trial the Fourier transform of the spike train via $c(\omega) = \frac{1}{T}\sum_{t_j}\exp(i\omega t_j)$ and consider its value at the frequency of interest, e.g. $\omega = 2\pi \cdot 11$ Hz in Fig. 2D. To calculate the distribution of the real and the imaginary parts of the Fourier coefficients $c(\omega)$ we calculate these values in each of N_T trials and pool their values across trials to construct the respective distributions in Fig. 2D and in the Supplementary Figs. 1-3B. For the amplitudes and the phases in Fig. 2E and the Supplementary Figs. 1-3C,D we subtract the trial averaged mean from each $c(\omega)$ in trials with repeating stimuli. To calculate the correlation coefficient in Fig. 2F and Supplementary Figs. 1-3E between the real and the imaginary values of $c(\omega)$ we calculate the product $\mathrm{Re}(c(\omega)) \cdot \mathrm{Im}(c(\omega))$ at each trial, sum them, divide by the number of trials and subtract the product of the trial averaged means before dividing by the product of the variances to obtain the correlation coefficient. Similarly, to evaluate the cross correlation of Fourier coefficients across frequencies in Fig. 2G in the Supplementary Figs. 1-3F we calculate at each trial $Re(c(\omega_1)) \cdot Re(c(\omega_2))$ sum them across trials, divide by the number of trials and subtract the product of the trial averaged means before dividing by the product of the variances to obtain the correlation coefficient. For the Fourier statistics displayed in Fig. 2 and the Supplementary Figs. 1-3 we set $N_T = 10.000$, $T = 40\,\mathrm{s}$ and voltage discretization step $dt = 0.1\,\mathrm{ms}$.

Numerical implementation of the direct method

Here, we describe the implementation of the direct method [17] which we used in our article. We implement this method to provide a quantitative comparison between the exact solutions of our correlation theory and a popular, currently used method for estimating the neural information content. At the core of the direct method is a discretization of each spike train, such that a "1" is assigned to a time bin of size T_{bin} if at least one spike occurred in this bin and a "0" otherwise. Each spike train is then partitioned into B bins, $T_{window} = B \cdot T_{bin}$. The word length B as well as the bin size T_{bin} has to be chosen such that the

length and structure of temporal correlations is well resolved and fully-contained in each word. The next step is the estimation of the occurrence probability for each of the possible 2^B binary patterns in a word consisting of B bins. To accurately estimate each of the 2^B probabilities it is necessary to have a sufficient number of observations for each of the patterns. Next, the probability of binary words $P(r)$ occurring during the N_T trials with varying stimuli and the probability of binary words $P(r|s)$ occurring during N_T trials of repeated stimulus presentations for each of $\#S$ stimuli are used to construct both the signal entropy $H_{signal} = \sum_R P(r) \log_2 P(r)$ and the noise entropy $H_{noise} = \frac{1}{\#S} \sum_S \sum_R P(r|s) \log_2 P(r|s)$, respectively, where the sums run over the 2^B different words and the $\#S$ different stimuli. Using the signal and noise entropies we obtain the mutual information as their difference $H_{signal} - H_{noise}$. Next, we follow the final steps detailed in [17] to obtain the information content and construct a linear fit of the information rate as a function of the inverse window length and evaluate its crossing point with the information axis at $1/T_{window} = 0$.

To choose the appropriate bin size, window lengths and trial number for a given data set, we make the following considerations. First, the bin size T_{bin} needs to be small enough to resolve the temporal structure of a spike train but also big enough to contain some spikes. Second, the word length $T_{bin} \cdot B$ needs to be at least as large as the longest temporal spike correlations such that one or more spikes can be observed and their interactions can be quantified. Particularly interesting in this context are non-trivial words. These are binary words which contain two or more spikes and which describe temporal spike interactions. Taking these considerations into account, we chose in Fig. 6 A,E-G bin size values such that they were below the temporal width of the respective auto correlation functions $C^{spike}_{auto}(\tau)$. Secondly, we chose the window lengths B such that $T_{bin} \cdot B$ was similar or larger than the temporal width of the respective correlation function $C^{spike}_{auto}(\tau)$ in order to capture the temporal structure of a spike train and allow for sizable non-trivial word probabilities. Let us note that spike trains where temporal correlations have a smaller range than the average interspike interval require small bin sizes and small to moderate window lengths. In this situation, the window size can be smaller than the average interspike interval and each word may have only few spikes. This is the regime where large N_T trial numbers are required to accurately estimate the occurrence of rare non-trivial words, words that contain more than one spike (see Supplementary Table 1). On the other hand, spike trains where the temporal correlations exceed an average interspike interval will typically need long window lengths and moderate bin sizes. In this situation, it is common to observe many spikes in a word and it is in general possible to estimate the occurrence probability of each binary word with moderate trial numbers because each of them occurs frequently. These considerations make it necessary to choose bin sizes, word lengths and trial numbers carefully for each data set. To show that our correlation theory provides an accurate estimate of information content across spiking models, firing rates and temporal structures we compared its results in Fig. 6 to the direct method in which we used the following parameters. In Fig. 6A,E,F we estimate the probabilities $P(r)$ using $N_T = 16 \cdot 10^6$ trials, in Fig. 6F(inset) $N_T = 64 \cdot 10^6$ and in Fig. 6G $32 \cdot 10^6$ trials (each containing a statistically independent stimulus) and $P(r|s)$ using $N_T = 0.5 \cdot 10^6$ trials in Fig. 6A,E,F,F (inset) and in Fig. 6G 10^6 trials for each of $\#S = 32$ stimuli ($\#S = 128$ for Fig. 6F (inset)). Further details of the direct method such as bin sizes, window sizes and the resulting non-trivial word statistics are summarized in the Supplementary Table 1.

Information in interspike intervals (ISI Information)

To estimate the information contained in the interspike interval distribution $P(ISI)$ and to contrast it with the complete information content of a spike train, we follow the procedure adapted from [19]. Here, we denote by ISI the interspike interval between two consecutive spikes t_1 and t_2 and aim to calculate the signal and noise entropies from $P(ISI)$ and $P(ISI|s)$. In order to calculate these quantities we need control of the stimulus and noise values between two consecutive spikes and to initialize the neuronal dynamics at t_1 with a controlled stimulus value. This initial value will require knowledge about the spike triggered distributions of the stimuli and the noise. Therefore, before proceeding with the calculation of signal and noise entropies we briefly comment on how to obtain these distributions. Spike triggered stimulus and noise distributions are obtained by starting with a random initial value and evolving the voltage, stimulus and noise dynamics as specified in the methods sections "input current statistics" and "Spiking neuron models". Specifically, this implies that at each time step of the Ornstein Uhlenbeck

current evolution the variable $\eta(t)$ is drawn randomly, see text to Eqs. 9-13. Similarly for binomial inputs, the stimulus and noise values at each time step are drawn randomly from the binomial distribution specified in Fig. 2. After a brief transient period, the voltage and spiking dynamics reaches a steady state where the respective spike triggered distributions can be obtained by measuring the distribution of stimulus and noise values at the spiking events.

Now, we proceed with the calculation of the signal entropy which is determined by $H_{signal}= \sum_{ISI} P(ISI)\log_2 P(ISI)$. Obtaining $P(ISI)$ involves repeating the following procedure for N_T trials. Starting at the time of a spike t_1, the voltage is initialized at the threshold value and the initial values for the noise and the stimulus are each drawn randomly from the respective spike triggered stimulus and noise distributions. Next, the voltage dynamics are evolved until the next spike t_2 using the dynamical equations of the respective neuron model and the stimulus and noise dynamics determined by Eqs. 9-13. The probability distribution $P(ISI)$ is then calculated from all N_T values of $t_2 - t_1$ via a histogram consisting of N_H bins. These bins are evenly distributed between zero and ten times the standard deviation of the considered ISI distribution. Let us note, that this procedure is equivalent to considering a long spike train emerging from the dynamics specified in Eqs.9-13 and by calculating $P(ISI)$ from N_T sequentially observed interspike intervals ISI_1 to ISI_N, because each interspike interval in this sequence will have naturally a different random initial signal and noise value.

Next, we address the calculation of the noise entropy which is given by $H_{noise} = \sum_s P(s) \sum_{ISI} P(ISI|s)\log_2 P(ISI|s)$. Obtaining $P(ISI|s)$ involves repeating the following procedure for N_T trials and each of $\#S$ different stimuli and then averaging over the stimuli. Starting at the time of a spike t_1, the voltage is initialized at the threshold value and the initial value for the noise is drawn randomly from its spike triggered stimulus distribution at each of N_T trials while the initial stimulus value is drawn randomly only on the first trial and kept frozen for the remaining $N_T - 1$ trials of a stimulus. Next, the voltage dynamics are evolved until the next spike t_2 using the dynamical equations of the respective neuron model and the stimulus and noise dynamics determined by Eqs. 9-13. At the first of N_T trials with a given stimulus, the sequence of stimulus values in time is drawn randomly but is kept frozen for the remaining $N_T - 1$ trials. The probability distribution $P(ISI|s)$ is then calculated from all N_T values of $t_2 - t_1$ values recorded for each of $\#S$ stimuli via a histogram consisting of N_H bins.

With the signal and noise entropies we only need to ensure that their difference, the mutual information $I(R, S)_{ISI}= H_{signal} - H_{noise}$, is precise enough for our choice of N_H. To this end, we evaluate the difference between the signal and noise entropies $I(R, S)_{ISI,N_H}$ as a function of N_H and study the regime $N_H = 10 - 10.000$ to make sure that $I(R, S)$ has converged towards its steady state. We find that the difference between signal and noise entropies reached its steady state for the spiking models in Fig. 5A at $N_H = 1000$ ($N_H = 5000$ for $\tau_{stim} = 0.2, 0.5$ ms) in Fig. 5B at $N_H = 5000$, for Fig. 5 A,B we used $N_T = 200.000$, $\#S = 320$.

Let us note that the complete information of a spike train is not an upper bound for the interspike interval information. Theorem 2.6.6 in [8] states that including temporal correlations will reduce both the signal and noise entropies, but they may not be reduced by the same amount. If the drop in noise entropy is larger than that of the signal entropy then their new difference can be larger than the original. To mechanistically describe when the ISI information over- or underestimates the full information, let's consider the numerator and denominator in equation (4). The numerator is the PSTH autocorrelation and therefore neglects any temporal ISI interactions capturing only rate covariation, see below. The spike auto correlation in the denominator is defined by two contributions, temporal interspike correlations and the distribution of interspike intervals $p(ISI)$. Neglecting temporal correlations within a spike train amounts to replacing $C^{spike}_{auto}(\omega)$ with that of a new process whose interspike intervals are independently drawn from $p(ISI)$ of the original spike process. Let's note that in contrast to the PSTH approximation, neglecting temporal correlations only *modifies* the auto correlation function but doesn't replace it with a delta function. For example if $p(ISI)$ is low for small ISIs, then the auto correlation function will exhibit a refractory period. Overestimation can occur if $C^{spike}_{auto}(\omega)$ is larger than can be expected from $p(ISI)$ alone. In this case, neglecting temporal correlations will primarily decrease the denominator in equation (4) and thereby increase the overall information content. Underestimation can occur in the opposite scenario where $C^{spike}_{auto}(\omega)$ is smaller than can be expected from $p(ISI)$ alone. The way temporal correlations and $p(ISI)$ combine to give rise to a spike auto correlation function is highly dependent

on the spike generation mechanism, noise and signal time scales, and therefore the amount of under- or overestimation may vary across neuron types and parameter details.

Supplementary Note 3

Additional details on the range of validity and the limits of correlation theory

Complex normal distributions obtained in integrate-and-fire type models for stationary, finite memory inputs (data in Fig. 6 E-G)

Here, we expand on our findings from the main manuscript Fig. 6 E-G and present evidence that the spikes of the leaky integrate-and-fire neuron, adaptive leaky integrate-and-fire neuron as well as of the exponential integrate-and-fire neuron, which we studied in this figure, indeed all show the signatures of independent complex Gaussian distributions. Parameter choices for each model as well as their Ornstein-Uhlenbeck input current statistics the neurons received can be found in the methods section of our main manuscript. In Fig. 6 E-G of the main manuscript we showed using these three models that the information content provided by our correlation theory matched the prediction of the direct method across two orders of magnitude of membrane time constants. Here, we provide further evidence in Figs. 1-3 on the Fourier statistics underlying this data. In panel A in Figs. 1-3 we show the voltage and spike trajectories as they emerge from the corresponding leaky integrate-and-fire neuron (Fig. 1 A), adaptive leaky integrate-and-fire neuron (Fig. 2 A) and the exponential integrate-and-fire neuron model (Fig. 3 A). We recognize that all three spike trajectories are largely irregular, where some spikes are emitted in close succession followed by a few isolated spikes. Let us note, that in the firing rate in the selected, short spike segments chosen for illustration can deviate from the average firing rate of this process, for reference the average firing rate is the caption to each figure. In panel B in Figs. 1 we show that the real parts of the Fourier coefficients at $f = 11\,\mathrm{Hz}$, $\omega = 22\pi\mathrm{Hz}$ ($f = 4\,\mathrm{Hz}$, $\omega = 8\pi\mathrm{Hz}$ in Figs.2- 3) for varying stimuli as well as repeated stimulus presentations follow Gaussian distributions, as can be expected from real and imaginary values of a complex normal distribution. Panel C in Figs. 1 - 3 shows, as predicted by our correlation theory, that the amplitudes of $c_R(\omega)$ and zero-centered $c_{R|s}(\omega)$ Fourier coefficients follow a Rayleigh distribution and have a uniform phase distribution (see panel D). In panels E, F in Figs. 1 -3 we recognize that in all three spiking models the real and imaginary parts of the Fourier coefficients $c_R(\omega)$ and $c_{R|s}(\omega)$ are largely independent and their correlation across frequencies vanishes.
In summary, Figs. 1-3 corroborate that the spike trains emerging from three integrate and fire neurons as they receive stationary finite memory inputs (Ornstein-Uhlenbeck in this example) fulfill all requirements of stationarity and finite memory at the spiking level and show all aspects of complex normal distributions in their Fourier coefficients that are required for the correlation theory to apply.

Additional examples exploring the limits of correlation theory via partially periodic processes

The correlation theory we derived in our manuscript is valid for spike trains that fulfill the assumptions of finite memory and stationarity. Here, we explore the limits of our correlation theory and show how it can gradually loose validity as the spike trains transition from stationary processes with finite memory to perfectly periodic, "clock"-like state that is neither stationary nor has finite memory. As this transition takes place, we will see that the distribution of Fourier coefficients transitions from a Gaussian to a multimodal distribution.
We start by considering a leaky integrate-and-fire neuron driven by an input current consisting of a stimulus and noise process that each alternate stochastically (Poisson switching with a rate $r = 10$ Hz) between two Ornstein-Uhlenbeck states (see 5 A). First, let us note that each of the two constituent Ornstein-Uhlenbeck states has finite memory therefore the combined switching process will have finite memory, too. We also know that each of the constituent processes is stationary, however their combination could potentially be non-stationary. To determine whether the combined switching process is stationary, we consider how its statistics evolve as a function of time. Following the definition, the interswitch intervals have a time invariant, exponential distribution. This means that the probability to jump from state 1 to state 2 is the same as vice versa, $p_{1\to 2} = p_{2\to 1} = r$, where r is the switching rate. Because it

is equally probable to leave state 1 as it is to leave state 2, the probability is equal to 0.5 for the process to be in one of the two states, and importantly, this probability is time invariant. The probability of a specific value s to occur is thus $P(s) = 0.5 \cdot p_{state_1}(s) + 0.5 \cdot p_{state_2}(s)$. This calculation indicates that the switching process is indeed stationary and has finite memory. It therefore fulfills the assumptions of our correlation theory. In Fig. 5A below we show an example of voltage trajectory and spikes in a leaky integrate and fire neuron driven by such a switching process. Fig. 5 B-E demonstrate that the spiking statistics in this model exhibit all signatures of a complex Gaussian predicted by our correlation theory, including the Gaussianity (Fig. 5 B), Rayleigh distribution of Fourier amplitudes (Fig. 5 C), independence of real and imaginary parts (Fig. 5 D) and independence across Fourier modes (Fig. 5 E). Additionally, we can confirm that the direct method by Strong, Koberle et al. [17] and our correlation theory yield equivalent results (Fig. 5F). For completeness, let us state the parameters we used in Fig. 5: Voltage threshold $V_{th} = 3$ mV, reset $V_{reset} = -2$ mV, stimulus correlation time $\tau_{stim} = 9$ ms; input variances $\sigma_{X,1} = 2.3$ mV and $\sigma_{X,2} = 3$ mV and stimulus-to-noise ratios $SNR_1 = 0.1$ and $SNR_2 = 0.8$.
Next, we consider a similar switching process but now replace the state 2 with a sinusoid. We consider a leaky integrate-and-fire neuron driven by an input current consisting of a stimulus and noise process (stimulus-to-noise ratio 0.6), each of which is a switching process which alternates stochastically between two states, state 1 and state 2. The current state 1 is an Ornstein-Uhlenbeck process and state 2 is a sinusoid. The life times of state 1 and state 2 follow the same exponential distribution and the switching times are Poissonian with a constant rate r. For concreteness we choose $r = 10$ Hz which results in an average state duration of 100 ms. State 1 is an Ornstein-Uhlenbeck process with a correlation time $\tau_{stim} = 10\,\text{ms}$ and an amplitude $\sigma = 1.46\,\text{ms}$. State 2 is a sinusoid of the form $I_{\sin}(t) = A_{\sin}\cos(2\pi \text{f} \cdot t + \phi)$, where the amplitude is $A_{\sin} = 12.5\,\text{mV}$ and frequency f = 50 Hz. As the processes switches from state 1 to state 2 the phase ϕ is randomly drawn at each onset from a uniform distribution, $\phi \in [-\pi, \pi]$. For concreteness we chose the following parameters for the leaky integrate and fire model. We set the threshold $V_{th} = 1$ mV, reset at -1 mV and membrane time constant at $\tau_{mem} = 25$ ms in 6 and 8. Now, lets address the stationarity and finite memory aspects of this stimulus process. Stationarity is met because the probability to be in state 1 or state 2 is constant across time and equal to 50%. The probability to obtain a given value $s(t)$ at a time t is given by the sum of probabilities describing each of the two states, each weighted by one half. The finite memory property is guaranteed by the random phase resets and the finite memory of state 1, the average time the system spends in state 2 introduces a periodic correlation structure lasting approximately 100 ms. We note that changing the average time the process spends in each of the two states or changing the statistics of segment lengths for each state will result in the same stationarity and finite memory outcome, as long as the phase coherence across periodic segments is lost after some finite amount of time.
Fig. 6 shows the resulting Gaussian statistics of the Fourier coefficients c_R and $c_{R|s}$. Fig. 6A demonstrates the variability across stimuli and three corresponding input currents (top,left) and a repeating stimulus (bottom, left) along side the corresponding spikes trains (right). In Fig. 6 B we confirm that the real parts of the Fourier coefficients are normal distributed. Fig. 6 C demonstrates that the amplitudes of $c_R(\omega)$ and $c_{R|s}(\omega)$ follow a Rayleigh distribution while their respective phases are uniform (inset). Also in Fig. 6, D we can confirm a lack of correlation between the real and imaginary parts of each Fourier coefficient and as well as across frequencies ranging from 1 Hz to 500 Hz. Small residual non-zero correlations remained due to finite size effects but these decreased with increasing recording time. These findings indicate that the statistics of the spike trains we considered show all signatures predicted for stationary and finite memory spikes, such that we can expect the correlation theory to be valid and correctly predict the neural information content. Indeed we confirm in Fig. 7 B that the information content predicted by our correlation theory closely matched the results of the direct method across multiple values of membrane time constants. In Figs. 7 A,C we show the expected decay of the spike auto and cross correlation functions for time delays beyond 100 ms and the corresponding decay of information as a function of frequency.
To further leave the validity regime of our correlation theory where stationarity and finite memory characterize the spike trains, we modified the input statistics to longer maintain phase coherence across all segments of state 2. We now select the phase ϕ at the transition point to state 2 such that the end of the previous state 2 segment and the beginning of the new state 2 segment maintain phase coherence. In other words, the phase ϕ is randomly selected at the beginning of each trial and remains constant such

that the same sinusoid $I_{\sin}(t) = A_{\sin}\cos(2\pi \mathrm{f}\cdot t + \phi)$ describes all state 2 segments within a recording. By opting for an infinitely long phase coherence across state 2, we mathematically break the assumption of finite memory and stationarity. Finite memory condition is broken due to infinitely long phase coherence and stationarity is broken because the probability of obtaining a specific value s in state 2 is now time dependent. This means that any information estimation procedure that assumes stationary spike distributions, such as the direct method or our correlation theory is no longer valid. However, in practice the spiking process still spends significant amount of time in the finite memory state 1 such that the finite and the infinite memory processes compete and both contribute to information coding. This means that both the direct method and our correlation theory are no longer exactly valid, and depending on how each one deals with the non-stationary contribution decides on how the information content will be altered. In the example we consider in Fig. 7 this could mean that the information values determined by the direct method and the correlation theory may coincide in some regimes while differ in others. We also note that the information content predicted by the correlation theory is largely unaffected by the non-stationary contribution (compare blue lines in Fig. 9 B and Fig. 9 B) while the information estimate provided by the direct method is strongly modified, particularly for short membrane time constants. Studying the statistics of the Fourier coefficients resulting from these stimuli in Fig. 8 A, we indeed obtain distributions that closely resemble Gaussian and Rayleigh statistics, see Fig. 8 B,C. However, the distribution of the correlation coefficients across frequencies in Fig. 8 D is still centered around zero, but its width is broader for trials with repeating stimuli. Furthermore, the Fourier statistics of this process indicate that in the regime of long time constants the amplitude of the coherent oscillations is much smaller than the peak of the cross correlation function, because the effect of the finite memory state 1 dominates (see Fig. 9). As a result we obtain a good correspondence between the direct method and our correlation theory. For smaller time constants, on the other hand, the amplitude of the periodic state 2 dominates and results in an increased periodic contribution to the cross correlation function (see Fig. 9 A).
We now transition to a counter example with infinitely long memory and non-stationary spike trains. In this counter example the periodic phase is infinitely long and is no longer interrupted by segments with finite memory processes (see Fig. 10 A-C). Therefore, the spike train is periodic with an infinitely long coherence function (see Fig. 10 A). As a result, the Fourier coefficients in Fig. 10 D-F are no longer Gaussian and their phases are no longer uniformly distributed (black solid lines serve as reference) but have a multimodal distribution that reflects the periodicity of the process (blue, red histograms). For concreteness, we chose in this example a mixture of periodic signals and noise processes, each described by $I_{\sin}(t) = A_{\sin}\cos(2\pi \mathrm{f}\cdot t + \phi)$, where $A_{\sin} = 45\,\mathrm{ms}$, frequency $\mathrm{f} = 50$ Hz and a signal-to-noise ratio of 0.6. The spiking threshold of the leaky integrate-and-fire model was set to $V_{th} = 3\mathrm{mV}$ and reset at $V_{reset} = -10\mathrm{mV}$ and membrane time constant was $\tau_{mem} = 15\,\mathrm{ms}$.

In summary, we have shown that the assumptions of our correlation theory gradually loose their validity as the input segments with an infinite memory gain prominence. In regimes where the finite memory contribution remains dominant the approximation provided by our correlation theory can remain accurate and provide an important reference (see Fig. 7). Delimiting the range of validity of our correlation theory can therefore be practically accomplished by studying the structure of the spike cross correlation function and its temporal extend and investigating the Fourier statistics with regard to independence and Gaussianity. Considering the fact that any biological process has finite life time and many intrinsic noise sources, it is plausible to assume that the finite memory property is met by a large class of recorded spike trains. Considering the fact that the global time evolution of sensory statistics and neural states can take place on a longer time scale than the intrinsic fine structure of the spikes, it is plausible to assume that stationarity can be a good approximation for time periods shorter than the global changes in sensory statistics.

References

[1] The computer code used in this study is available from `www.tchumatchenko.de/code_snarticle.zip`.

[2] P. Dayan and L. F. Abbott. *Theoretical Neuroscience: Computational and Mathematical Modeling of Neural Systems.* The MIT Press, 2001.

[3] D. R. Brillinger. *Time series: data analysis and theory*, volume 36. Siam, 2001.

[4] D. R. Brillinger. Fourier analysis of stationary processes. *Proceedings of the IEEE*, 62(12):1628–1643, 1974.

[5] T. Kawata. On the fourier series of a stationary stochastic process. *Probability Theory and Related Fields*, 6(3):224–245, 1966.

[6] D. R. Brillinger. The spectral analysis of stationary interval functions. *Selected Works of David Brillinger*, pages 25–55, 2012.

[7] M.E. Taylor. *Measure Theory and Integration.* American Mathematical Society, 2006.

[8] T. M. Cover and J. A. Thomas. *Elements of Information Theory* . Wiley-Interscience, 2nd edition, 2006.

[9] H. Kobayashi, B. L. Mark, and W. Turin. *Probability, Random Processes, and Statistical Analysis: Applications to Communications, Signal Processing, Queueing Theory and Mathematical Finance.* Cambridge University Press, 2011.

[10] J. V. Michalowicz, J. M. Nichols, and F. Bucholtz. *Handbook of differential entropy.* CRC Press, 2013.

[11] R. B. Stein, A. S. French, and A. V. Holden. The frequency response, coherence, and information capacity of two neuronal models. *Biophysical journal*, 12(3):295–322, 1972.

[12] A. Borst and F. E. Theunissen. Information theory and neural coding. *Nature neuroscience*, 2(11):947–957, 1999.

[13] F. Gabbiani. Coding of time-vaying signals in spike trians of linear and halfwave rectifying neurons. *Network Comput. Neural Sys.*, 7:61–65, 1996.

[14] F. Rieke, D. Warland, R. D. Steveninck, and W. Bialek. *Spikes: Exploring the Neural Code.* The MIT press, 1997.

[15] O. Schwartz, J. W. Pillow, N. C. Rust, and E. P. Simoncelli. Spike-triggered neural characterization. *Journal of Vision*, 6(4):484–507, 2006.

[16] J. S. Bendat and A.G. Piersol. *Random data: analysis and measurement procedures*, volume 729. John Wiley & Sons, 2011.

[17] S. P. Strong, R. Koberle, R. R. de Ruyter van Steveninck, and W. Bialek. Entropy and information in neural spike trains. *Phys Rev Lett*, 80(1):197–200, 1998.

[18] A. Renart, J. de la Rocha, P. Bartho, L. Hollender, N. Parga, A. Reyes, and K.D. Harris. The asynchronous state in cortical circuits. *Science*, 327:587–590, 2010.

[19] B. N. Lundstrom and A. L. Fairhall. Decoding stimulus variance from a distributional neural code of interspike intervals. *The Journal of Neuroscience*, 26(35):9030–9037, 2006.
