# Supplementary material for: Temporal pairwise spike correlations fully capture single-neuron information
Source: Nat Commun. 2016 Dec 15;7:13805. doi: 10.1038/ncomms13805 (PMC5171810; doi:10.1038/ncomms13805)
Supplement: Supplementary Software — This supplementary file contains computer code underlying the results of the manuscript and it includes an implementation of the correlation-based information estimate, ISI-based information and the direct method. Within we provide also an additional file examplecode-fcreconstruction.zip to help understand how Gaussian Fourier statistics can emerge in stationary spike trains when Fourier coefficients are pooled across multiple trials. [file ncomms13805-s2.zip › examplecode-fcreconstruction.zip!/ExampleCode_FCReconstruction/Fig1_SpikeReconstruction.pdf]

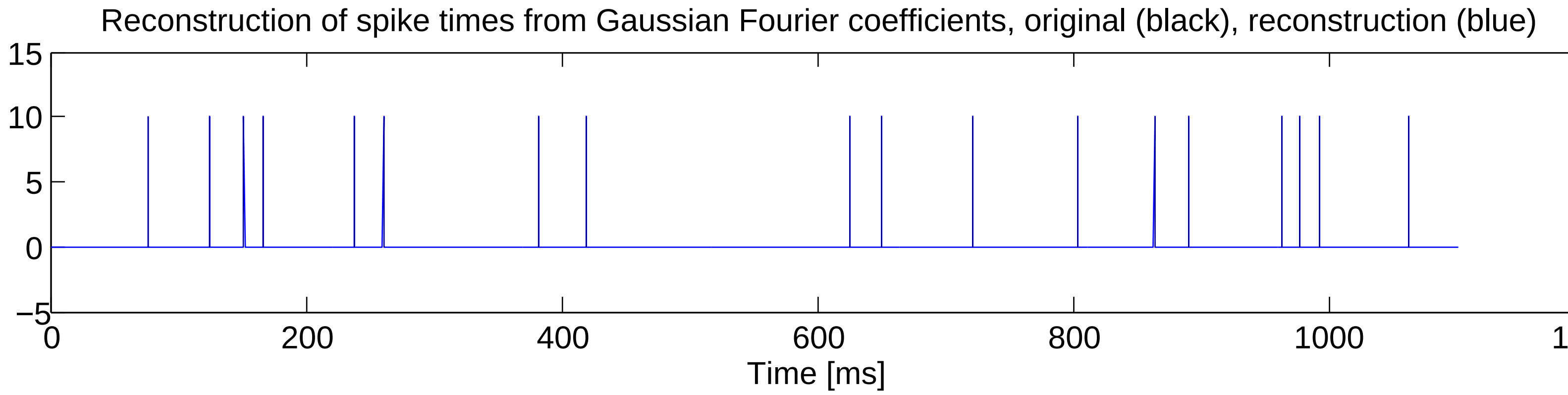
Reconstruction of spike times from Gaussian Fourier coefficients, original (black), reconstruction (blue)
15
10
5
0
−5
0
200
400
600
800
1000
Time [ms]
