# Supplementary material for: Temporal pairwise spike correlations fully capture single-neuron information
Source: Nat Commun. 2016 Dec 15;7:13805. doi: 10.1038/ncomms13805 (PMC5171810; doi:10.1038/ncomms13805)
Supplement: Supplementary Software — This supplementary file contains computer code underlying the results of the manuscript and it includes an implementation of the correlation-based information estimate, ISI-based information and the direct method. Within we provide also an additional file examplecode-fcreconstruction.zip to help understand how Gaussian Fourier statistics can emerge in stationary spike trains when Fourier coefficients are pooled across multiple trials. [file ncomms13805-s2.zip › examplecode-fcreconstruction.zip!/ExampleCode_FCReconstruction/Fig2_GaussianFourierCoefficients.pdf]

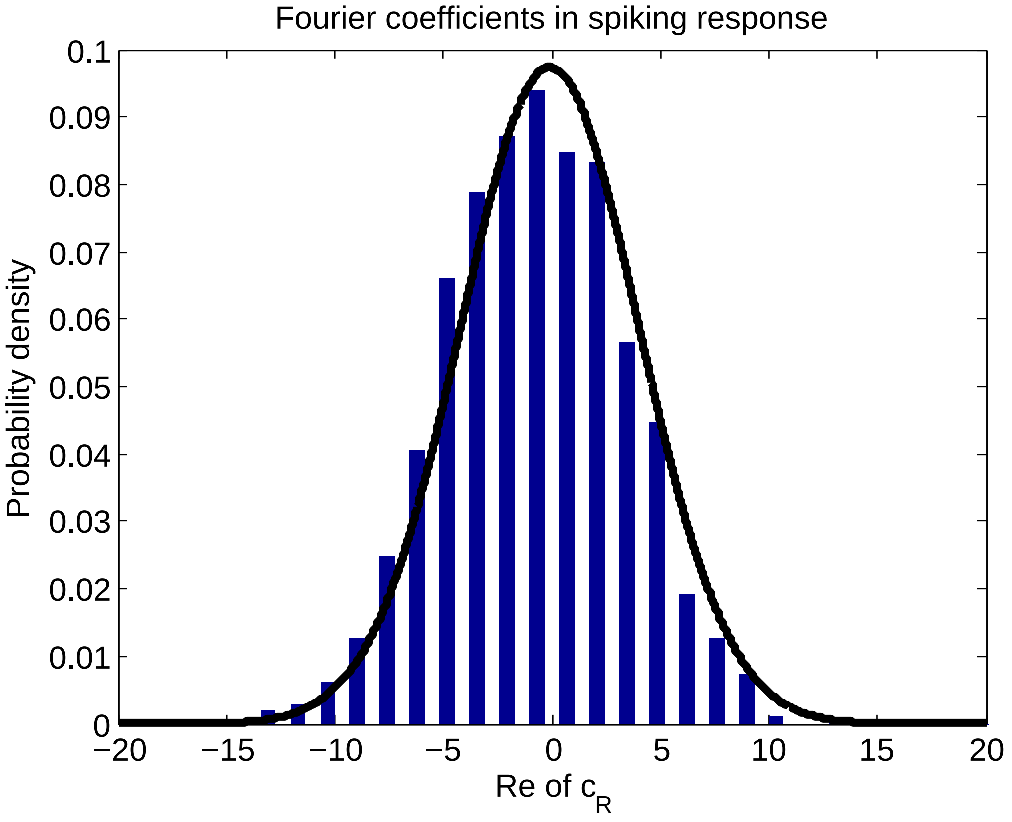

Fourier coefficients in spiking response
Probability density
Re of c_R
0.1
0.09
0.08
0.07
0.06
0.05
0.04
0.03
0.02
0.01
0
−20
−15
−10
−5
0
5
10
15
20
